# Effects of Calcium Spikes in the Layer 5 Pyramidal Neuron on Coincidence Detection and Activity Propagation

**DOI:** 10.3389/fncom.2016.00076

**Published:** 2016-07-22

**Authors:** Yansong Chua, Abigail Morrison

**Affiliations:** ^1^Institute for Advanced Simulation (IAS-6), Theoretical Neuroscience and Institute of Neuroscience and Medicine (INM-6), Computational and Systems Neuroscience, Jülich Research Center and Jülich Aachen Research AllianceJülich, Germany; ^2^Faculty of Biology, Albert-Ludwig University of FreiburgFreiburg im Breisgau, Germany; ^3^Bernstein Center Freiburg, Albert-Ludwig University of FreiburgFreiburg im Breisgau, Germany; ^4^Institute for Infocomm Research, Agency for Science, Technology and Research (A^*^STAR)Singapore, Singapore; ^5^Faculty of Psychology, Institute of Cognitive Neuroscience, Ruhr-University BochumBochum, Germany

**Keywords:** calcium spikes, layer 5 pyramidal neurons, coincidence detection, activity propagation, synfire chains, detailed balance, short term plasticity

## Abstract

The role of dendritic spiking mechanisms in neural processing is so far poorly understood. To investigate the role of calcium spikes in the functional properties of the single neuron and recurrent networks, we investigated a three compartment neuron model of the layer 5 pyramidal neuron with calcium dynamics in the distal compartment. By performing single neuron simulations with noisy synaptic input and occasional large coincident input at either just the distal compartment or at both somatic and distal compartments, we show that the presence of calcium spikes confers a substantial advantage for coincidence detection in the former case and a lesser advantage in the latter. We further show that the experimentally observed critical frequency phenomenon, in which action potentials triggered by stimuli near the soma above a certain frequency trigger a calcium spike at distal dendrites, leading to further somatic depolarization, is not exhibited by a neuron receiving realistically noisy synaptic input, and so is unlikely to be a necessary component of coincidence detection. We next investigate the effect of calcium spikes in propagation of spiking activities in a feed-forward network (FFN) embedded in a balanced recurrent network. The excitatory neurons in the network are again connected to either just the distal, or both somatic and distal compartments. With purely distal connectivity, activity propagation is stable and distinguishable for a large range of recurrent synaptic strengths if the feed-forward connections are sufficiently strong, but propagation does not occur in the absence of calcium spikes. When connections are made to both the somatic and the distal compartments, activity propagation is achieved for neurons with active calcium dynamics at a much smaller number of neurons per pool, compared to a network of passive neurons, but quickly becomes unstable as the strength of recurrent synapses increases. Activity propagation at higher scaling factors can be stabilized by increasing network inhibition or introducing short term depression in the excitatory synapses, but the signal to noise ratio remains low. Our results demonstrate that the interaction of synchrony with dendritic spiking mechanisms can have profound consequences for the dynamics on the single neuron and network level.

## 1. Introduction

Calcium spikes have been extensively studied in many *in-vitro* experiments (Stuart et al., 1997; Larkum et al., 1999b,a, 2001; Hay et al., 2011; Almog and Korngreen, 2014) and their influence on neuron firing activities has been investigated in *in-vivo*-like simulation settings (Larkum et al., 2004, 2009; Shai et al., 2015). As an outcome of this research, the calcium spike has been hypothesized as the main biological mechanism to help propagate synaptic inputs at the distal tuft to the soma of large layer 5 pyramidal neurons, which would otherwise have little influence on the membrane potential at the soma, and thus on the neuron's spiking activity (Larkum et al., 2009). Two biological mechanisms thought to be of particular importance are back-propagating action potential triggered calcium spikes Larkum et al. (1999a), where the active back propagation of action potentials to the distal dendrites combined with a coincident distal input triggers a calcium spike that in turn triggers a burst of action potentials, and critical frequency, in which three or more pulses of synaptic inputs or currents delivered to the soma at sufficiently high frequency trigger calcium spikes, and thus further action potential bursts Larkum et al. (1999b). Hence, calcium spikes have been proposed to be critical for coincidence detection (Spruston, 2008), and the binding of synaptic inputs from various brain regions (feedforward sensory inputs on the basal dendrites and feedback attentional inputs on the distal tuft) in the layer 5 pyramidal neuron which is also the output neuron of the mammalian neocortex (Larkum, 2013). Shai et al. (2015) also propose that the calcium spike could be important for orientation tuning.

While it is clear that dendritic spikes play an important part in the execution of cognitive tasks, for example by enabling or enhancing stimulus response and selectivity (Sivyer and Williams, 2013; Smith et al., 2013; Grienberger et al., 2014; Palmer et al., 2014), the contribution of calcium spikes to neural network computation, and the *in-vivo* role of the biological mechanisms identified in the *in-vitro* studies mentioned above, has remained as an area of ongoing research. For instance, Traub and Wong (1982) showed that hippocampal neurons with intrinsic bursting properties synchronize to generate epileptic activities. More recently, both theoretical and experimental studies suggest that calcium spike mediated activity propagation in feed-forward networks (FFN) could underlie the neural sequence generation in HVC neurons of the singing zebra finches (Jin et al., 2007; Long et al., 2010). Dendritic spikes with constant waveforms have also been investigated with respect to their effect on network dynamics (Memmesheimer, 2010; Jahnke et al., 2012; Memmesheimer and Timme, 2012). There, the model was first devised using fairly general considerations before being applied to fast dendritic sodium spikes, which have been shown to reproduce experimental results in hippocampal networks (Memmesheimer, 2010; Jahnke et al., 2015) and to enhance activity propagation (Jahnke et al., 2013, 2014). On the topic of slow dendritic spikes, modeling work carried out by Lisman et al. (1998) suggests that slow NMDA spikes help sustain neural activities critical for maintenance of working memory and slow dendritic spikes in general increase the memory capacity of recurrent neural networks (Breuer et al., 2014).

As for theoretical work in modeling the calcium spike, while simulations have been carried out at single neuron level using NEURON (Carnevale and Hines, 2006), the amount of background noise applied may not have been sufficient to emulate the *in-vivo* situation. Also, quantitative measures have yet to be be proposed for coincidence detection. Hence, while calcium dynamics has been shown to increase frequency of firing with increased basal and distal coincident inputs (Shai et al., 2015), change of firing activities may also be achieved by the coincident inputs alone, i.e., without calcium spikes. This provides the motivation to quantify coincidence detection of the neuron while receiving coincident inputs, with and without calcium dynamics. Moreover, to our knowledge, there are few, if any, network studies that investigate how calcium spikes at single neuron level interact with the network dynamics. While previous work has addressed activity propagation enhanced by fast dendritic spikes (Jahnke et al., 2013, 2014), the slow calcium spike has properties that deserve further investigation. One such property is that calcium spikes last for tens of milliseconds and have a long plateau peak, bringing the distal membrane potential close to the excitatory reversal potential, hence severely attenuating the effect of nearby excitatory synaptic inputs (Larkum et al., 1999a). In addition, synaptic inputs impinge on the large pyramidal neurons with varying electrotonic distance to the soma, which in turn interact with the calcium spike in a differentiated manner, potentially enhancing activity propagation in a neural network of spatially extensive neurons differently as well. The long duration of the calcium spike and the compartmentalization of the layer 5 pyramidal neuron thus introduce network effects not accounted for using an exponential current in point neuron models; the effect of calcium spike dynamics on the propagation of firing activities in a network therefore remains an open question.

To address the above issues, we have first developed a three compartment neuron model of the layer 5 pyramidal neuron (Chua et al., 2015) and showed that the calcium spike can be modeled using first order kinetics or as a threshold triggered fixed waveform when the neuron is in a low fluctuation driven regime, with calcium spikes triggered by occasional large coincident inputs of a fixed time constant, which mimics the *in-vivo* situation. This model is summarized in Section 2.1. Here, we systematically investigate the behavior of the neuron model in such a regime. In particular, we are concerned with determining the effect of calcium spikes on the detection of coincidence inputs and propagation of firing activities in a FFN embedded in a random network, with a particular focus on whether the coincident input arrives solely at the distal synapses or are shared between the distal and somatic compartments. The single neuron and network simulations are described in Section 2.2 and the corresponding measures for coincidence detection and activity propagation are given in Section 2.3.

In the single neuron scenario, we determine that the presence of calcium spikes does confer an advantage in reliable and informative spike detection if the coincident inputs only impinge on the distal dendrite, but not if they also impinge on the soma (Section 3.1.1 and Section 3.1.2). Interestingly, the default parametrization of our neuron model does not reproduce the critical frequency phenomenon observed in Larkum et al. (1999b); therefore our results demonstrate that this mechanism is not a vital part of the coincidence detection mechanism as previous argued (Larkum et al., 1999b; Shai et al., 2015). Further, using a minor variant of the model parametrization that does reproduce the critical frequency behavior, we demonstrate that this phenomenon disappears in the presence of *in-vivo* like background noise.

In a network scenario (Section 3.2), we discover that where excitatory neurons are connected to each other only at the distal compartment, calcium spikes are necessary for activity propagation in an embedded FFN, which remains stable across a large range of synaptic strengths. In the case of connections at both somatic and distal compartments, the presence of calcium spikes reduces the minimum width of a FFN capable of supporting activity propagation. However, networks with stronger synapses become unstable, as the activity propagation interacts with spontaneous synchrony in the network. The instability can be compensated by increasing network inhibition or using exc-exc synapses with depressing short term plasticity (Tsodyks et al., 1998, 2000).

Our results thus emphasize the importance of calcium spikes in detecting and efficiently propagating coincidences, but suggest that the critical frequency phenomenon is not an important mechanism for neural processing.

## 2. Materials and methods

To determine how calcium spikes in the layer 5 pyramidal neuron may enhance its ability to detect and propagate coincident action potentials, we developed a three compartment neuron model in Chua et al. (2015). We provide the model with fluctuating synaptic inputs and quantify its ability to detect and propagate coincident action potentials. We describe the neuron models and their parametrization in Section 2.1, and the variations of synaptic inputs that the neuron receives in Section 2.2. In Section 2.3, we introduce the analysis of spiking activities used to quantify our simulation results.

### 2.1. Neuron models

**Figure 1:**
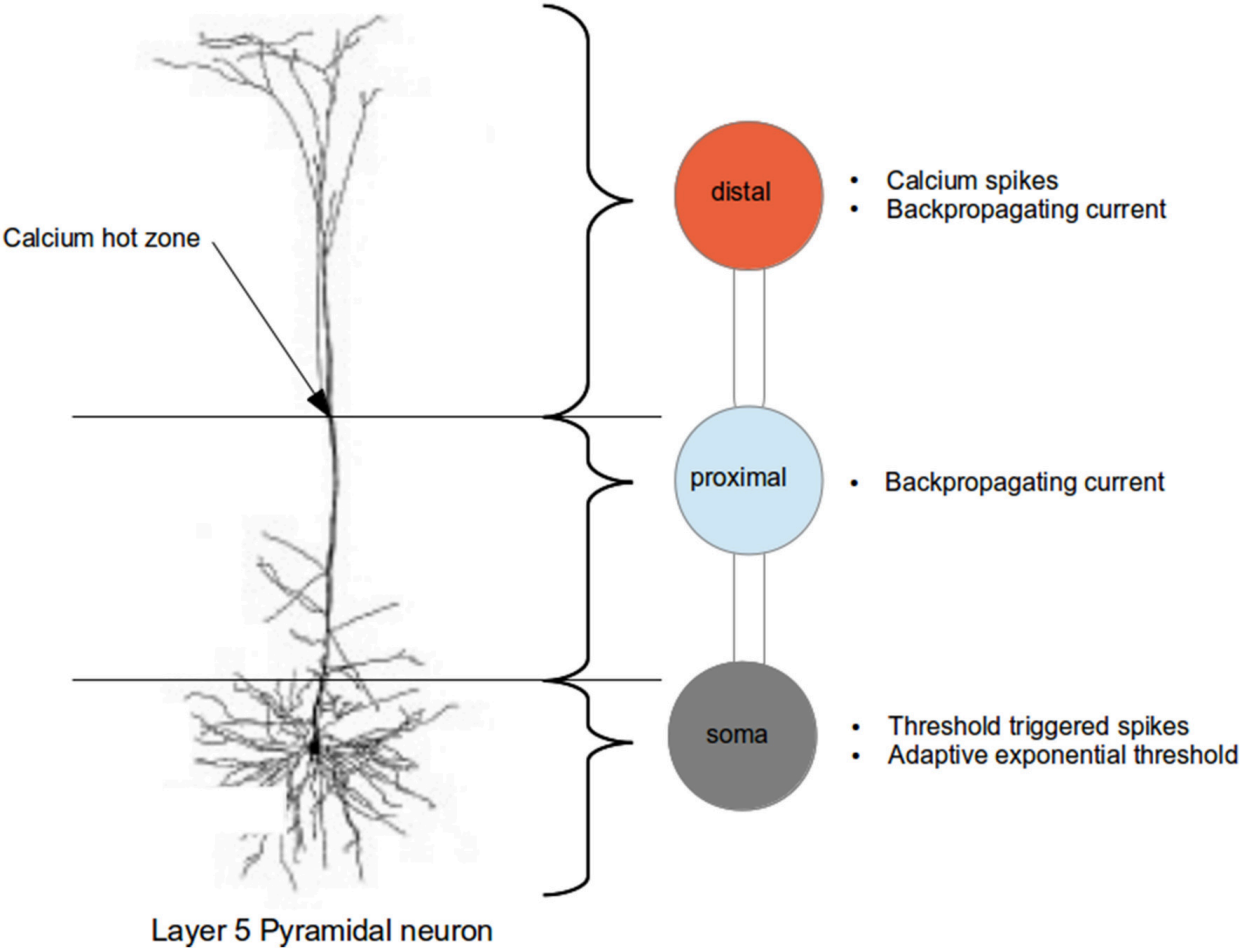
**Schematic of the three compartment model of the layer 5 pyramidal neuron indicating the regions abstracted by each compartment and their dynamical properties (compared with actual neuron, from Spruston, 2009)**.

We represent the layer 5 pyramidal neuron as a system of three connected isopotential compartments (see Figure 1), with dynamics described by three coupled first order differential equations governing the time evolution of the membrane potentials of the three compartments

(1) $$\begin{array}{l} \left(\begin{array}{l} C^{\text{d}}\;{\dot{V}}^{\text{d}}\\ C^{\text{p}}\;{\dot{V}}^{\text{p}}\\ C^{\text{s}}\;{\dot{V}}^{\text{s}} \end{array}\right)\;=\;(\begin{array}{l} \;-\sum_{x\in \{l,e,i\}}g_{x}^{d}(V^{d}-U_{x}^{d})+\;I_{ca}\\ \text{​​​​}-\sum_{x\in \{l,e,i\}}g_{x}^{p}(V^{p}-U_{x}^{p})+g_{\text{pd}}\left(\left(V^{\text{d}}-U_{\text{l}}^{\text{d}})-(V^{\text{p}}-U_{\text{l}}^{\text{p}}\right)\right)\\ \;-\sum_{x\in \{l,e,i\}}g_{x}^{s}(V^{s}-U_{x}^{s}) \end{array}\\ \begin{array}{l} \;+g_{\text{pd}}\;\left(\left(V^{\text{p}}-U_{\text{I}}^{\text{p}})-(V^{\text{d}}-U_{\text{I}}^{\text{p}}\right)\right)+\;I_{\text{Ap}}^{\text{d}}\\ \;+g_{\text{sp}}\left(\left(V^{\text{s}}-U_{\text{I}}^{\text{s}})-(V^{\text{p}}-U_{\text{l}}^{\text{P}}\right)\right)+\;I_{\text{Ap}}^{\text{d}}\\ \;+g_{\text{sp}}\;\left(\left(V^{\text{p}}-U_{\text{l}}^{\text{p}})-(V^{\text{s}}-U_{\text{l}}^{\text{s}}\right)\right) \end{array}) \end{array}$$

where the superscripts d, p, and s denote the distal, proximal and somatic compartments, respectively. *C* and *V* refer to capacitance and membrane potential and *g*_*l*_ is the leak conductance, whereby the resting potential is equal to $U_{l}^{y}$ when there are no external inputs. The constants *g*_pd_ and *g*_sp_ are the conductances across the distal-proximal and soma-proximal compartments, and *U*_e_ and *U*_i_ are the excitatory and inhibitory reversal potentials. The calcium current *I*_ca_ is modeled using first order kinetics or threshold triggered waveform in the distal compartment, as proposed in Chua et al. (2015).

As explained in greater detail in Chua et al. (2015), a three step approach was taken to fit the parameters of the neuron model. In the first step, only the neuron parameters such as capacitance and leak conductances are fitted; calcium dynamics is deactivated in this step. An exhaustive search for the above parameters within biologically realistic range is performed so that a step current applied at the soma produces an action potential as per Figure 1C of Larkum et al. (1999a), and a hyperpolarizing current at the proximal followed by a beta current at the distal do not trigger any action potential as per Figure 5C2 of Larkum et al. (2001). Next, the calcium dynamics is activated, and we fit the calcium parameters (of first order kinetics) such that they are able to reproduce calcium spike and action potentials as per Figures 1D,E of Larkum et al. (1999a). In the final step, we increase the action potential threshold dynamically by adjusting the parameters in the adaptive threshold so as to reduce the number of calcium spike triggered action potentials to the numbers observed experimentally.

When embedded in low rate background noise, we discovered that a calcium spike could be triggered in the neuron by 400 spikes arriving simultaneously at the distal dendrite (with synaptic weight of 0.6 nS each). Synchrony however does not need to be precise; a calcium spike could still be triggered by the spikes arriving with a delay normally distributed with σ = 6ms more than 90% of the time, before falling off to 0 calcium spikes as σ increased to 10.

The neuron model parametrized as above does not reproduce the critical frequency (CF) property demonstrated by Larkum et al. (1999b) and Shai et al. (2015): three step input currents applied at the soma (each triggering an action potential) of sufficient high frequency trigger a calcium spike at the distal compartment, resulting in additional bursts of action potentials. However, by modifying a minimal set of parameters, as listed in Table 1 in the Appendix (Supplementary Material), we are also able to reproduce this property.

### 2.2. Simulation protocols

The neuron models are simulated in three main protocols:

- The single neuron receives low fluctuating inputs [as per Tables 2, 3 in the Appendix (Supplementary Material)] with occasional large coincident inputs at just the distal, or both soma and distal compartments.
- The single neuron receives regular step current stimulation at the soma with or without low fluctuating inputs. Fluctuating input details are described in Tables 2, 3 in the Appendix (Supplementary Material).
- The neurons are arranged in a topological recurrent network, and a fraction of the excitatory neurons are stimulated at the somatic and/or distal compartments with synaptic inputs from other neurons in the network.

#### 2.2.1. Single neuron with occasional coincident inputs in noisy background

The regime of interest for this study is where the neuron is receiving mostly low fluctuating inputs with occasional coincident inputs, such that its firing rate is approximately 1 spikes/s and the average membrane potential at the soma is approximately −60 mV. These requirements are slightly relaxed in simulation settings (described below) in which the synaptic weights are lognormally distributed and synaptic inputs have an oscillating firing rate. To investigate under which circumstances calcium spikes are triggered and in turn lead to action potential bursts, we vary the amount of coincident inputs.

To create coincident input, the spike train arriving at each synapse is composed of two superimposed Poisson spike trains. The first spike train contains the synchronous input, and is formed by copying each spike from an original spike train (also known as the mother process, since inputs copy from this same spike train Kuhn et al., 2003) with a certain copy probability *p*, which ranges from 0.01 − 0.5. As we define the mother process has a spike rate of 1 spike/s, each child spike train has a spike rate of *p* spike/s. We require the total input at each synapse to have a rate of 1 spike/s, so an additional asynchronous spike train is drawn with Poisson statistics and rate 1 − *p* spike/s. The superposition of the synchronous and asynchronous spike trains result in a Poisson spike train of rate 1 spike/s for each synapse, such that the pairwise correlation between any two coincident input is *p* (Kuhn et al., 2003).

There are three further aspects of the synaptic inputs to be considered:

##### 2.2.1.1. Location of coincident inputs

Coincident inputs either impinge on 30% of excitatory synapses in the distal compartment (distal inputs), or 20% of excitatory synapses in the distal compartment and 10% of excitatory synapses in the soma compartment (shared inputs). We have also simulated 15% of excitatory synapses in each of the distal and somatic compartments, as opposed to 20/10.

##### 2.2.1.2. Synaptic weight distribution

Excitatory synaptic weights are either identical or drawn from a lognormal distribution. In the lognormal case, we assigned coincident inputs to the synapses with the heaviest weights. Given a lognormal weight distribution with a mean equal to the identical weight, its variance will cause increased fluctuation in the conductances and an increased firing rate, even in the case where there are no coincident inputs. As we keep a constant ratio of the standard deviation of the weight to its mean, we have to adjust the mean synaptic weight downwards to keep the neuron's firing rate close to 1 spike/s. As a result of this adjustment, the somatic mean free membrane potential is slightly below −60 mV. The adjustment is made using mean firing rate and membrane potential values obtained from simulations of the base case whereby there are no coincident inputs.

#### 2.2.2. Single neuron with regular step current stimulation

Three step currents (of amplitude 1500 pA, duration 2ms, and frequency ranging from 10 to 200 hz frequency) are applied at the somatic compartment at regular interval of every 1000ms. In the case of additional background noise, it is generated by synaptic Poisson input (2000 and 500 excitatory and inhibitory Poisson inputs with spike rates of 1 spike/s and synaptic weights of 0.6 nS and 1.0 nS at each compartment respectively), resulting in low fluctuations and a spike rate of 1 spike/s [see Tables 2, 3 in the Appendix (Supplementary Material)].

#### 2.2.3. Feed-forward chains in recurrent network

An excitatory population (8100 neurons, modeled using the three compartment neuron model with either first order kinetics or fixed waveform calcium spike), and an inhibitory population (2025 leaky-integrate-and-fire neurons with conductance based synapses) are each placed regularly on a 2D torus grids of the same size and recurrently and reciprocally connected. The connection probability is given by a 2D Gaussian distribution, centered at the location of the post-synaptic neuron (Mehring et al., 2003). Network parameters are chosen to result in an asynchronous irregular firing regime at low rate [see Table 4 in the Appendix (Supplementary Material)].

We next embed a FFN (see, e.g., Diesmann et al. 1999) consisting of a series of pools of neurons, such that synchronous activity in the first pool propagates along the embedded network (Vogels and Abbott, 2005; Kumar et al., 2008). We assign both excitatory and inhibitory neurons to corresponding pools in the same proportions that they exist in the embedding network, along the diagonals of the respective unrolled tori. This results in two superimposed FFNs of nine pools with 100 neurons in each pool (for distal inputs) or 15 pools with 36 excitatory neurons each (for shared inputs).

Note that this embedding strategy differs from that implemented by Jahnke et al. (2012), in which neurons in the first pool are picked randomly, and each neuron in the subsequent pool selected based on number of connections with neurons in previous pool. In our study, neurons are assigned to their respective pool according to their location on the grid.

The excitatory neurons are connected to each other either only at the distal compartment so as to emulate the distal inputs case, or at both the somatic and distal compartments so as to emulate the shared inputs case.

To facilitate propagation of activity, the synaptic weights in the FFN are strengthened by a scaling factor; in all cases, excitatory-excitatory connections (between two consecutive pools in the excitatory FFN) are strengthened; in for the Gaussian shared case, all four connection types are strengthened (excitatory-excitatory connections between consecutive pools in the excitatory FFN, excitatory-inhibitory connections between one pool in the excitatory FFN and the next pool in the inhibitory FFN, inhibitory-excitatory between one pool in the inhibitory FFN and the next pool in the excitatory FFN and inhibitory-inhibitory connections between consecutive pools in the inhibitory FFN) so as to achieved detailed balance (Vogels and Abbott, 2009).

Input spikes are then applied to excitatory neurons in the first pool of the excitatory FFN only. This is repeated five times, with interval of 2.25 s apart. The excitatory connections between pools are then multiplied by an increasing scaling factor in subsequent simulations and activity propagation again measured to assess effect of increased synaptic weights. For a full listing of the network parameters, see Table 4 in the Appendix (Supplementary Material).

In addition, we also perform single neuron simulations similar to those described in Section 2.2.1, but adapted to provide the neuron with input corresponding to that received by a neuron in a FFN. To approximate the input received by a neuron in a distally connected FFN, an excitatory synaptic input is applied at the distal compartment, with synaptic weight multiplied by two factors, namely the scaling factor and the expected number of synaptic inputs from the prior excitatory pool, on top of the background noise from Poisson inputs. To approximate the input received by an excitatory neuron in a FFN with shared connectivity, an excitatory synaptic input is applied at the distal and somatic compartments, with synaptic weight multiplied by two factors, namely the scaling factor and the expected number of synaptic inputs from the prior excitatory pool. In addition, it also concurrently receives an inhibitory input at the soma, so as to reproduce the detailed balance in the network setting. The Poissonian firing rates vary at different compartments as the average network firing rates obtained empirically differ from the original input rate of 1 *spikes*/*s* chosen in Section 2.2.1. The synaptic weights are chosen identically to those in the network simulations. For a full listing of the network parameters, see Table 3 in the Appendix (Supplementary Material).

### 2.3. Measures and analysis

#### 2.3.1. Coincidence detection for single neuron simulation

We employ two measures for quantifying the spike coincidence detection properties of the neuron model: reliability and informativity, as defined below. The reliability of the response indicates how likely it is that a coincident input event triggers the neuron to generate an action potential (or burst of action potentials). Conversely, the informativity of the response indicates how likely it is that an action potential (or burst) was preceded by a coincident input event. A neuron is a good coincidence detector when reliability and informativity are both high. If reliability is high but informativity low, coincident events trigger spike bursts, but so does random synaptic noise. If informativity is high but reliability low for bursts of a given size, then a burst of that size indicates a prior coincident event, but there are also many other coincident events that do not trigger a burst. The different scenarios are summarized in Table 1. As the above measures are based on how the neuron spiking activities change with coincident inputs. we have generalized coincidence detection to also include coincidence propagation.

**Table 1:** **Reliability and informativity**.

		**Informativity**	
		**Low**	**High**
**Reliability**	**Low**	Only a small fraction of coincident inputs triggers a given neuronal response, and a large fraction of the neuronal responses is triggered by fluctuating inputs	Only a small fraction of coincident inputs triggers a given neuronal response, but a large fraction of the neuronal responses is triggered by coincident inputs
	**High**	A large fraction of coincident inputs trigger a given neuronal response, but a large fraction of the neuronal responses is triggered by fluctuating inputs	A good coincidence detector: a given neuronal response occurs if and only if there is a coincident input

##### 2.3.1.1. Reliability

**Figure 2:**
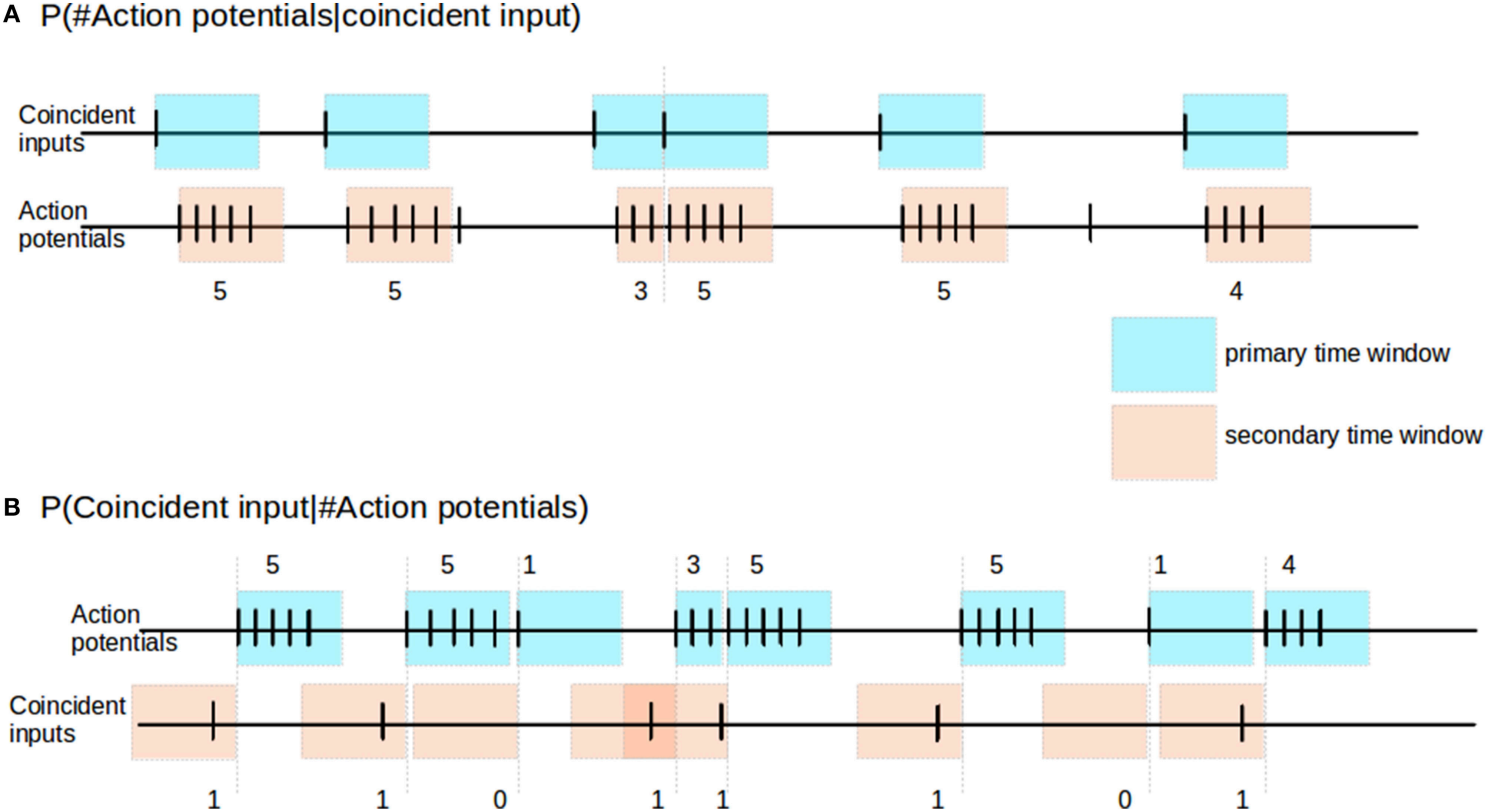
**Reliability and informativity**. **(A)** Schematic illustrating measurement of reliability. Blue shaded areas denote primary time window triggered by the occurrence, pink shaded areas denote secondary time window, triggered by the occurrence of an action potential within the primary time window. The response of the neuron is measured as the number of action potentials within the secondary time window. **(B)** Schematic illustrating measurement of informativity. Blue shaded areas denote primary time window, triggered by the occurrence of an action potential; the burst size of the neuron is defined as the number of action potentials falling in this window, as indicated above it. Pink shaded areas denote secondary time windows stretching back in time from the start of each primary window, and indicate the relevant period for searching for a preceding coincident input.

The reliability of the neuronal response can be inferred from the statistics of the number of action potentials occurring in the short time window immediately after a coincident input event. This measure is illustrated in Figure 2A. Following each coincident input *C*, we define a primary time window (blue) of 100 ms or until the next coincident input, whichever is smaller. We identify any action potentials of the output spike train falling within this window. In the case that an action potential exists, we then mark out from this first action potential a secondary time window (pink) of 100 ms or until the next coincident input, whichever is smaller. We then count the number of action potentials *a* falling in the secondary time window. Over the course of an experiment we thus empirically obtain the distribution of action potential counts given a coincident input, i.e., *P*(*a*|*C*), where *a* ∈ {0, 1, 2, 3, ≥ 4}.

##### 2.3.1.2. Informativity

The informativity of the neuronal response can be expressed as the probability of a coincidence event given a certain action potential count. To determine this we proceed as illustrated in Figure 2B. We first look for occurrences of action potentials in the spike train of the neuron. Given an action potential, we count the total number of action potentials *a* within a primary time window (blue) of 100 ms or until the next coincident input, whichever is smaller. We then use a secondary time window (pink) of 100 ms to look back in time from the first action potential to determine whether there was a coincident input *C*. Hence, from the above, given a particular action potential event, if there is no coincident input in its secondary time window, then the informativity value for the action potential event is 0. Over the course of an experiment we thus obtain empirically the distribution of coincident inputs given a particular action potential count, i.e., *P*(*C*|*a*).

By construction, informativity is the posterior probability of reliability. Hence from Bayes theorem, the reliability *P*(*a*|*C*) can be computed as such:

(2) $$P(a|C)=\frac{P(C|a)N_{a}}{Nc}$$

where *N*_*a*_ is the total number of bursts of size *a* and *N*_*C*_ is the total number of coincident events. The time window used in this study is 100 ms; using 50 ms yields similar results (results not shown). Hence, reliability values obtained analytically using Equation (1) serve to verify the correctness of the empirically obtained reliability values.

#### 2.3.2. Activity propagation in network simulation

The measure used to quantify activity propagation between pools of the FFN for different scaling factors is the signal-to-noise ratio (SNR) as described in Jahnke et al. (2012). Here, signal refers to the spiking activity, which is defined by the number of action potentials of the neurons in a particular pool measured within a specific time window (here 100 ms, as spiking activities propagate from one pool to the next after an initial strong stimulus is introduced to the first pool. Noise refers to spiking activity of the neurons in the pools when no such stimuli was introduced. Hence a high SNR is achieved only if the background activity is sparse compared to the spiking activity propagating along the FFN following a stimulus. The SNR is computed for every pool after each stimulus applied to the first pool of the FFN, and then averaged across trials to obtain a final SNR value for each pool and each scaling factor.

## 3. Results

### 3.1. Neuronal response to background noise

**Figure 3:**
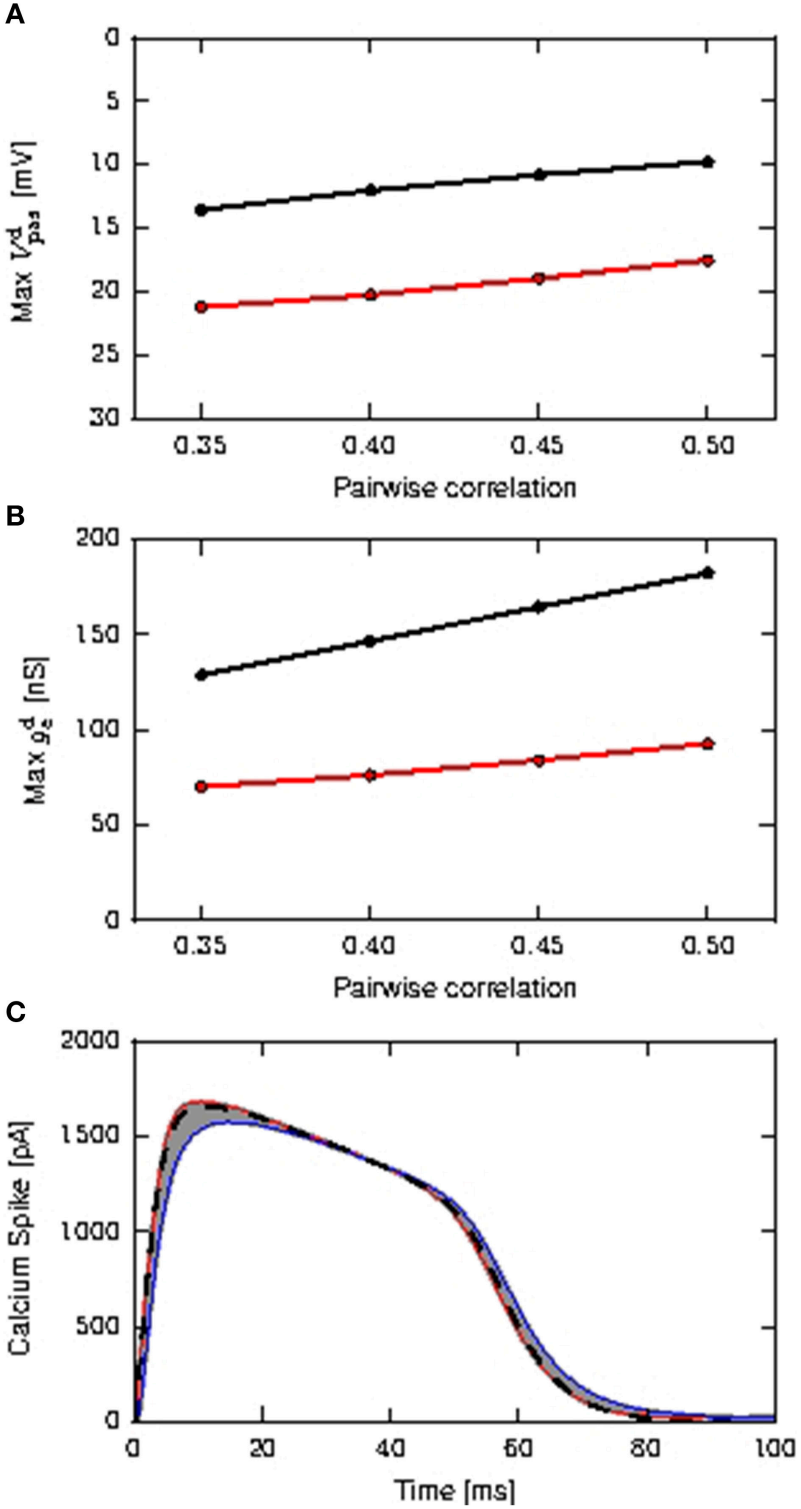
**Determining the properties of the calcium spike for purely distal and shared distal and somatic inputs**. **(A)** Maximum amplitudes of distal EPSP obtained from the passive neuron model, as a function of the pairwise correlation of the coincident input, distal inputs (black) and shared inputs (red). **(B)** Maximum amplitudes of the mean distal excitatory conductances during calcium spikes as a function of the pairwise correlation of the coincident input; color scheme as **(A)**. **(C)** Calcium spikes (first order kinetics) triggered by the coincident inputs for τ_e_ = 1.0ms. The red curve is the average calcium spike triggered by distal coincident inputs with pairwise correlation of 0.5, while the blue curve is the average calcium spike triggered by shared coincident inputs with pairwise correlations of 0.35. All other average calcium spikes fall within the gray area. The black dashed curve denotes the calcium waveform used for the fixed waveform model, obtained from averaging across all calcium spikes.

As a prerequisite for our investigation into the effectiveness of the calcium spike in coincidence detection of coincident inputs on both somatic and distal compartment, we first determine if the waveform and threshold need to be modified from the case where coincident inputs arrive only at the distal compartment, for the case where coincident inputs arrive at both the somatic and distal compartments. As presented in Chua et al. (2015), the threshold of the calcium fixed waveform can be obtained empirically from the maximum distal membrane potential reached by the input EPSP (Figure 3A). Using the method represented in Figure 6B of Chua et al. (2015), we determine a calcium threshold is −21.4 mV for distal inputs and −23.6 mV for shared inputs.

To understand why the thresholds are different, we first note that the calcium threshold is determined by the dynamics of the first order kinetics model, and is thus influenced by both the amplitude and shape of the EPSP—a slower decaying potential requires a lower amplitude to trigger the calcium spike (Chua et al., 2015). In the case that all coincident input arrives at the distal compartment, the EPSP is higher but also faster decaying than for shared inputs, where some of the input goes to the soma and can thus only affect the distal EPSP after being diminished by the leak conductance, as shown in Figures 3A,B. Hence the distribution of the coincident input to one or two compartments of the model causes different calcium spike triggering EPSPs, resulting in different determinations of the corresponding threshold. However, the fixed waveform of the calcium spikes used in the reduced model is obtained from the average calcium spike modeled using first order kinetics, as shown in Figure 3C, and so is the same in both scenarios.

These values serve as a basis for our investigation of the role of location of synapses receiving coincident input in determining the effectiveness of calcium spikes in triggering an action potential, or burst of them, as described in Section 3.1.1 and Section 3.1.2. In addition, we examine the effects of two other important aspects of neuronal input: the synaptic weight distribution and the temporal structure of the stimulus.

Synaptic weights have been shown to be lognormally distributed; the standard deviation to mean ratio of the lognormal distribution is 1.18, as calculated by Song et al. (2005). To investigate how lognormally distributed synaptic weights might affect calcium spike-action potential dynamics, we simulate both identical and lognormally distributed synaptic weights. We further assume that the lognormal synaptic weight distribution arises as a result of the interaction of coincident input with STDP, and therefore assign the coincident input stimulus to the synapses with the heaviest weights.

With respect to the temporal structure of the input to the neurons, it has been shown that cortical pyramidal neurons have up and down states Holcman and Tsodyks (2006). Excitatory synaptic inputs arrive not at a stationary rate but at rates modulated by some oscillatory frequencies van Kerkoerle et al. (2014). Our results for fluctuating inputs with oscillating firing rates are however, not qualitatively different from the case of stationary synaptic inputs, and hence we only show results from the simpler case.

#### 3.1.1. Distal coincident inputs in stationary noise

**Figure 4:**
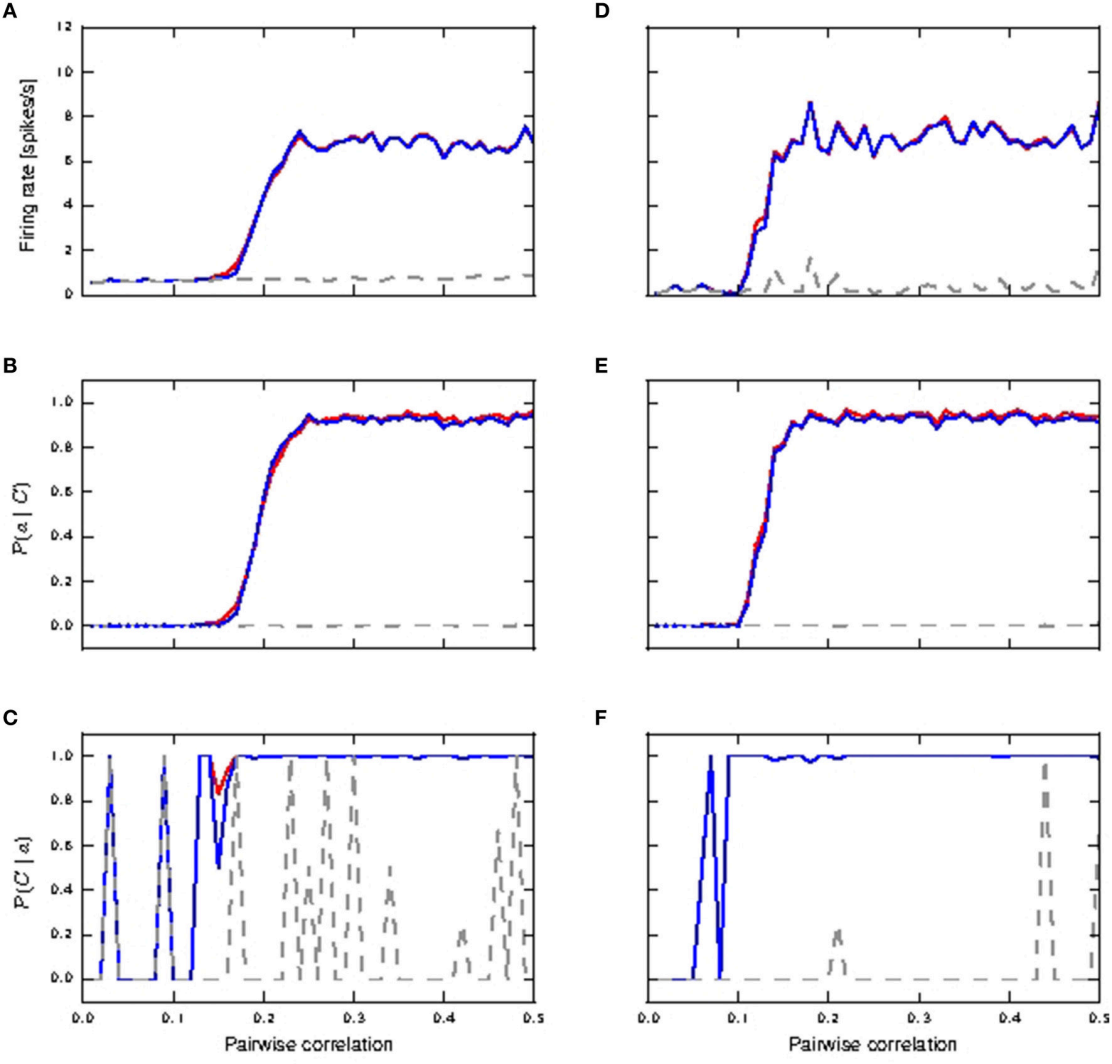
**Neuronal response to distal coincident inputs superimposed on stationary Poisson noise as functions of the pairwise correlation of the input with identical synaptic weights (A–C) and lognormally distributed weights (D–F)**. **(A,D)** Firing rate of passive neuron model (gray), neuron model with fixed waveform (blue) and with first order kinetics (red). **(B,E)** Reliability: probability of a burst event of ≥4 action potentials following a coincident input; colors as in **(A)**. Reliability values as computed directly from simulation results are shown as solid curves while reliability values computed using informativity simulation values as per Equation (1) are shown using markers. **(C,F)** Informativity: probability of a preceding coincident input given a burst of ≥ 4 action potentials; colors as in **(A)**.

Figure 4 shows the response of all three considered neuron models to distal coincident inputs with stationary Poisson input, for both identical and lognormally distributed synaptic weights. For low pairwise correlation, all three models behave much the same. The fixed waveform model is identical to the passive model in this regime, as the coincident input is not enough to trigger a calcium spike. The first order kinetics model still produces small currents for the lower coincidences, but this has a negligible effect on the neuron behavior. In both weight distribution scenarios, the neuron models with active responses to coincident input at a higher pairwise correlation (calcium spikes modeled by either kinetics or fixed waveform), have similar firing rates of 6–8 *spikes*/*s*, whilst the passive neuron remains at around 1 spike/s (see Figures 4A,D).

Action potential bursts of ≥4 spikes are reliably triggered by coincident inputs from pairwise correlation value of around 0.25 onwards, with the lognormal case doing so at slightly smaller pairwise correlations (see Figures 4B,E). Smaller burst events are not reliably triggered at any correlation value. Reliability results computed from simulation and those analytically derived from informativity simulation results using Equation (1) are in agreement, and so are omitted in the rest of this study. At the same time, these action potential events of the active neuron are highly informative to its postsynaptic neurons of pre-synaptic coincident events to its distal compartment (see Figures 4C,F). Note that a value of 0 in Figures 4B–F can also indicate that there were no events of the given burst size. Thus, the fluctuating curves in Figures 4C,F for the passive neuron, and for the active neuron models at low pairwise correlation, indicates that bursts of ≥4 action potentials occurred extremely rarely, but always after a coincident input.

Hence, the presence of a calcium spike in a neuron receiving background noise and distal coincident inputs considerably increases its coincidence detection properties, with bursts of ≥4 action potentials as the event of interest. In contrast, the passive neuron (without calcium spike) receiving the same synaptic inputs fares badly for both measures.

#### 3.1.2. Distal and somatic coincident inputs in stationary noise

**Figure 5:**
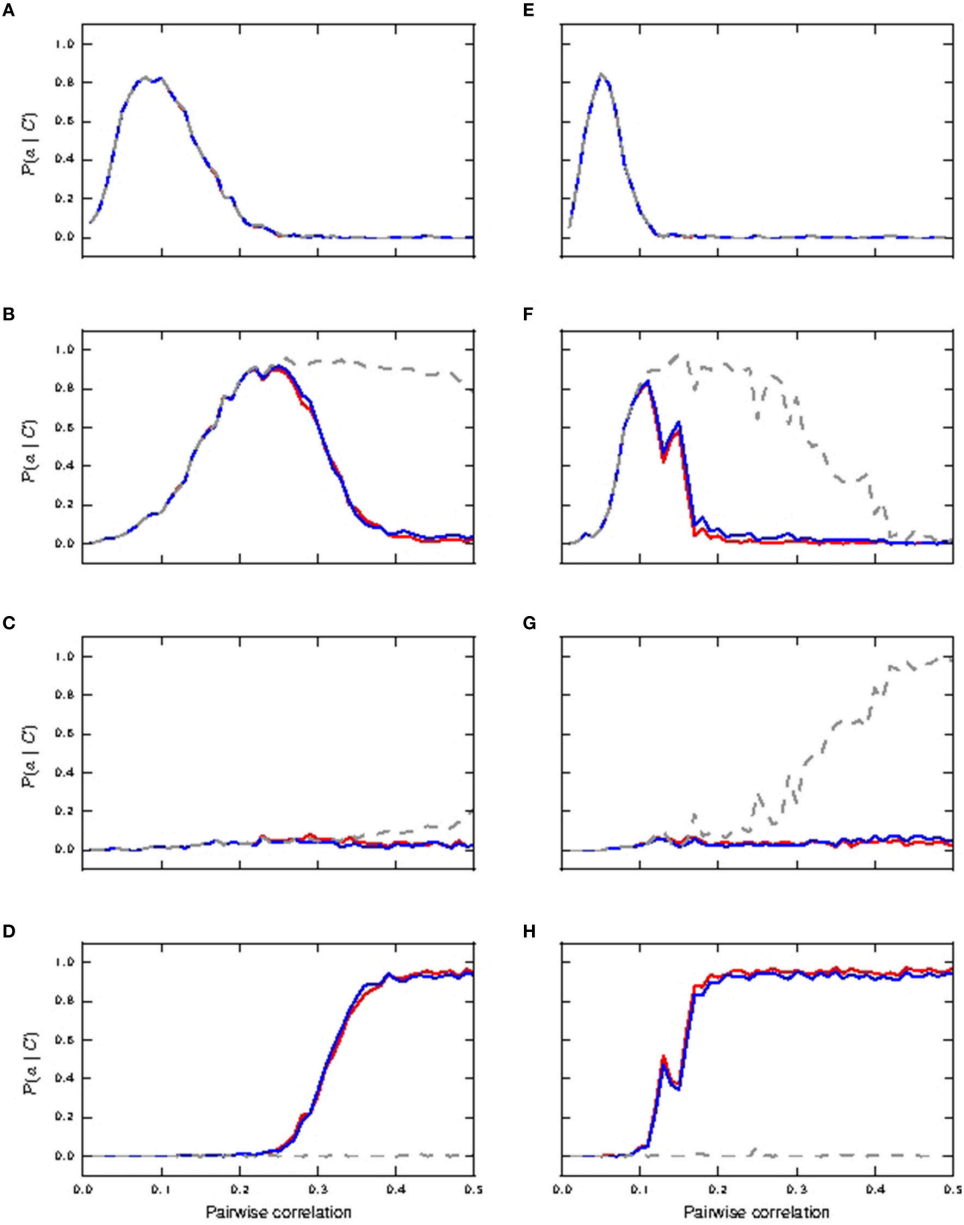
**Reliability of neuronal response to shared coincident inputs (15% in somatic and distal compartments) superimposed on stationary Poisson noise as functions of the pairwise correlation of the input with identical synaptic weights (A–D) and lognormally distributed weights (E–H)**. **(A,E)** Reliability results for single action potential; passive neuron model (gray), fixed waveform (blue), and first order kinetics (red). **(B,F)** Reliability results for burst of size two. **(C,G)** Reliability results for burst of size three. **(D,H)** Reliability results for burst of size ≥4.

We next investigate the scenario that coincident inputs arrive at both the distal and the somatic compartments (15% each compartment). Figure 5 shows how reliably the coincident inputs can trigger bursts of different magnitudes. In the case that all synaptic weights are identical, we observe that bursts of size two or more can be very reliable from a pairwise correlation of around 0.2 (Figure 5B) for passive and active neuronal dynamics, and bursts of size three are not reliably triggered for either the passive or the active neuron model (Figure 5C). Whereas bursts of size two continue to be highly reliable for the passive neuron for higher pairwise correlation values, for the active neuron models these bursts are subsumed in bursts of size four from pairwise correlation values of around 0.35 onwards (see Figure 5D). Note that bursts of size two are due to somatic coincident inputs and not due to calcium spikes: they do not occur for the active neuron models when the coincident input is applied solely to the dendritic compartment (see Section 3.1.1), and they do occur for shared input for the passive neuron. This demonstrates that calcium spikes are only reliably triggered by coincident inputs from this point onwards. As coincident inputs are shared across the somatic and distal compartments, the amount of coincident inputs at the distal compartment is halved. Comparing to the distal case, in which bursts of size ≥4 are reliably triggered at a pairwise correlation of around 0.25 (see Figure 4B), we may conclude that for the fluctuating regime, while there is cooperativity of coincident inputs across compartments in generating calcium spikes (and hence bursts), they are less effective in doing so than a focused distal input.

In the lognormal case, Figures 5E,F show that single action potentials are reliably subsumed in bursts of size two at a lower pairwise correlation compared to the response of the neurons with identical synaptic weights. These remain a reliable indicator in the passive neuron model until the bursts of size two are subsumed in bursts of size three at a pairwise correlation of around 0.4 (Figure 5G). For the active neuron models, the bursts of size two are subsumed in bursts of size ≥4 from a pairwise correlation of around 0.2 (see Figure 5H), whilst bursts of size three hardly ever occur. Reliability results calculated from informativity using Equation (1) are not shown, as they give identical results as reliability computed from simulation data.

**Figure 6:**
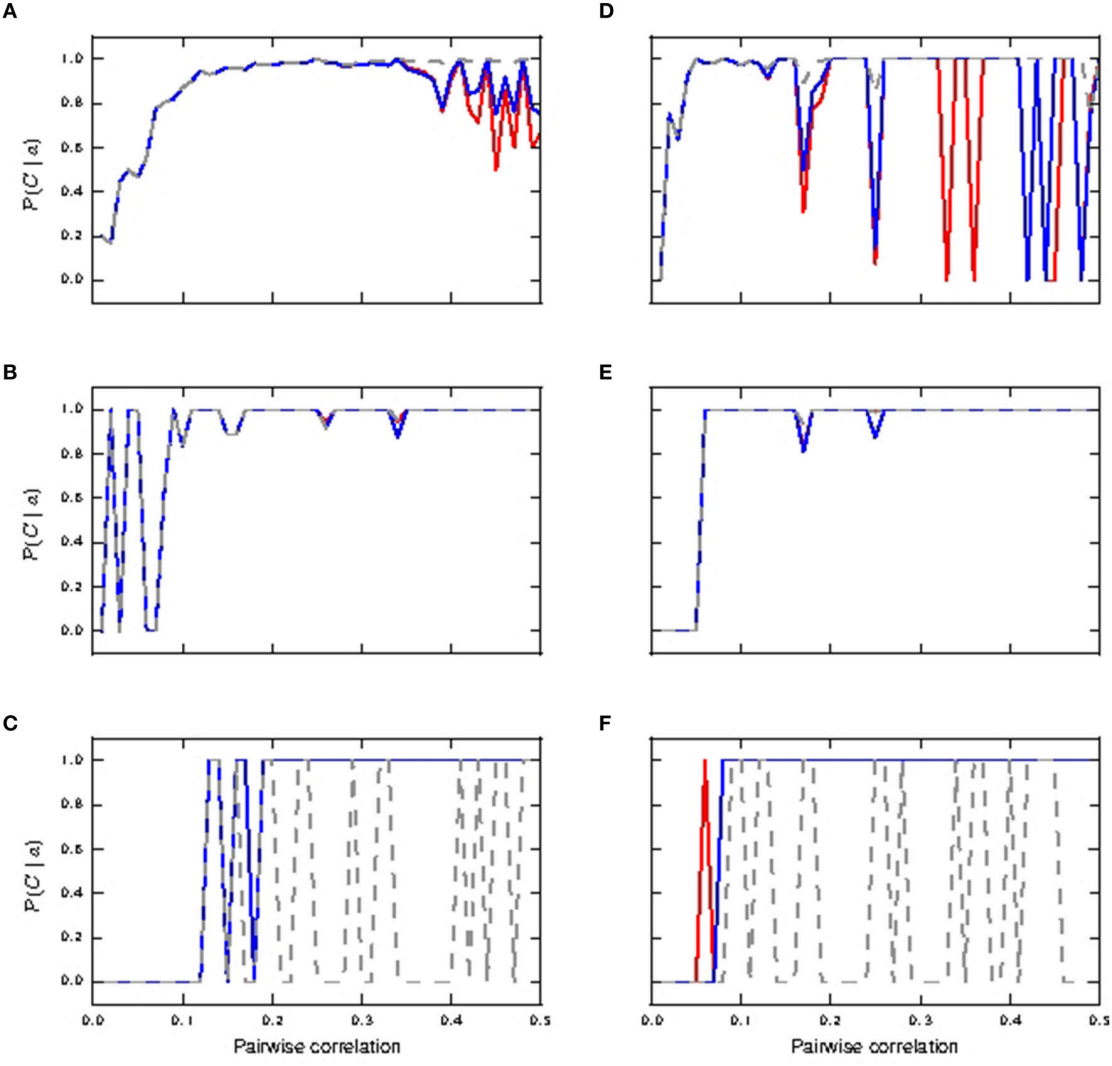
**Informativity of neuronal response with respect to shared coincident inputs (15% in somatic and distal compartments) superimposed on stationary Poisson noise as functions of the pairwise correlation of the input with identical synaptic weights (A–C) and lognormally distributed weights (D–F)**. **(A,D)** Informativity of bursts of size two; passive neuron model (gray), fixed waveform (blue), and first order kinetics (red). **(B,E)** Informativity of bursts of size three. **(C,F)** Informativity of bursts of size ≥4.

Figure 6 shows the informativity of different burst magnitudes in signaling a preceding coincident event. Bursts of size two are informative of a prior coincident input in the passive neuron model from pairwise correlation value ≈ 0.1 onwards for both identical and lognormally distributed synaptic weights, while its informativity in the active neuron model drops off with increased coincident inputs (Figures 6A,D). Bursts of size three are highly informative in all cases from correlation value ≈ 0.1 onwards (Figures 6B,E). However, we note that they are only reliably triggered in the passive neuron from correlation value ≈ 0.4 onwards in the lognormal case and hardly at all in the uniform case (see Figures 5C,G).Bursts of size ≥4 are only informative for the active neuron neuron models, as they are seldom triggered in the passive neuron model, which can be observed from the fluctuating values (see Figures 6C,F). As with Figures 4C,F, the fluctuating curves indicate that bursts of size ≥4 were very rare, but were always informative of a preceding coincident event.

We can conclude that in the identical weight case, bursts of size two accurately detect coincidences for active and passive neuron models in the range of mid pairwise correlation values and for high correlation values in the passive neuron model, while bursts of size ≥4 become more accurate for the active neuron model at high correlation values. For the lognormal case, bursts of size ≥4 makes the active neuron model a good coincidence detector across both pairwise correlation ranges. These results are summarized in Table 2. Hence, calcium spikes only play a role in coincidence detection in the identical weight case from high pairwise correlation values onwards, while they play a role at much lower values for the pairwise correlation in the lognormal case.

**Table 2:** **Size of bursts that are reliably informative for coincidence detection in the shared input case**.

		**Identical**		**Lognormal**	
		**Passive**	**Active**	**Passive**	**Active**
Pairwise correlations	Mid (0.2 ≤ *x* < 0.35)	2	2	2	≥4
	High (0.35 ≤ *x* ≤ 0.5)	2	≥4	3	≥4

The results in this and the previous section additionally demonstrate that the neuron model employing a threshold triggered fixed waveform (Chua et al., 2015) is in very close agreement with the neuron model employing first order kinetics for background noise with superimposed coincident inputs. Therefore, for the remainder of the manuscript we restrict our analysis to the responses of the neuron model with a threshold triggered fixed waveform, unless otherwise stated.

**Figure 7:**
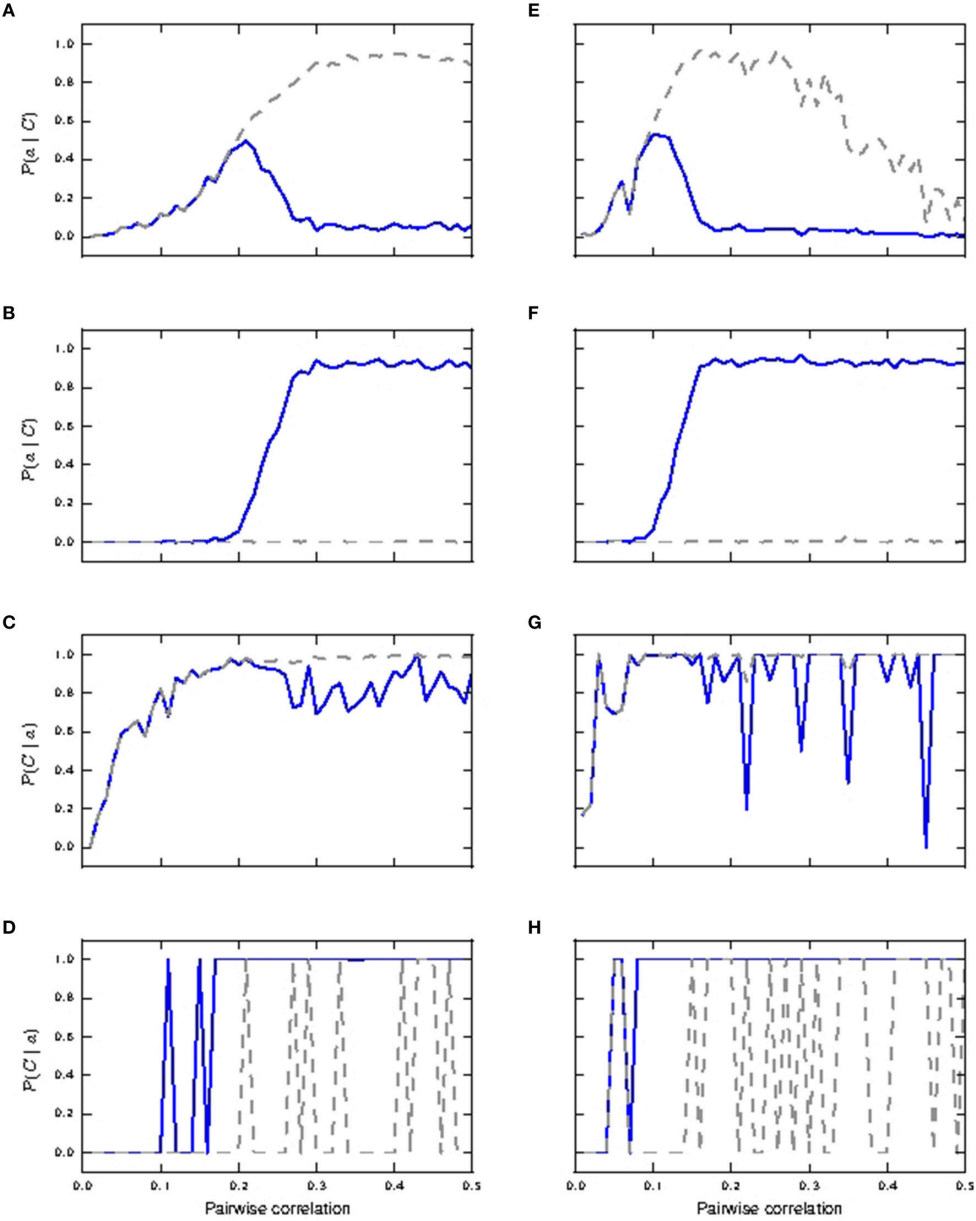
**Reliability and informativity results of neuronal response to shared coincident inputs (10% in the somatic and 20% in the distal compartment) superimposed on stationary Poisson noise as functions of the pairwise correlation of the input with identical synaptic weights (A–D) and lognormally distributed weights (E–H)**. **(A,E)** Reliability for burst of size two: passive neuron model (gray) and fixed waveform (blue). **(B,F)** Reliability for bursts of size ≥4. **(C,G)** Informativity for burst of size two. **(D,H)** Informativity for bursts of size ≥4.

We next investigate the effects of shifting the distribution of the coincident inputs to predominantly distal. Figure 7 depicts the case of shared coincident inputs arriving at 10% of the excitatory synapses onto the soma and 20% of those onto the distal compartment. For synapses with identical weights, bursts of size two are reliably triggered in the passive neuron from a pairwise correlation value of around 0.3 (Figure 7A) when a single action potential is subsumed in bursts of size two, as compared to 0.2 for an equal input distribution (Figures 5A,B); no bursts of higher magnitude are generated (Figure 7B) in the passive case. The main difference to the case where the coincident inputs are equally distributed is that bursts of size two are never reliably triggered in the active neuron, and bursts of size three are not triggered at all; these smaller magnitude responses are subsumed in bursts of size ≥4, which are reliably triggered from a lower correlation value (compare Figures 7B, 5D).

With lognormally distributed synaptic weights, the main difference from the equal input distribution case of 15% coincident inputs to the somatic and distal compartments is again that bursts of size two are never reliably triggered in the active neuron (Figure 7E). The smaller magnitude response is subsumed in response of bursts of size ≥4, which become reliable at a lower pairwise correlation value than in the equal input distribution case (Figure 7F).

The difference arises because the predominantly distal coincident input is more effective at triggering calcium spikes, which in turn generate bursts of size ≥4 at lower correlations than the equal input distribution. In the absence of the calcium spike, the increasing correlation tends to generate bursts of size 2, which are somatically driven, as is clear from their occurrence in the passive neuron model.

Together with informativity results in the identical weight case (Figures 7C,D), bursts of size two and burst of size ≥4 make the passive and active neurons respectively good coincidence detectors from pairwise correlation values ≈ 0.3 onwards. In the lognormal weight case (see informativity results Figures 7G,H), burst of size two (and at higher pairwise correlation values, burst of size three; not shown) and burst of size ≥4 make the passive and active neurons respectively good coincidence detectors from pairwise correlation values ≈ 0.15 onwards. Therefore, in contrast to the case in which coincident inputs arrive solely at the distal compartment (Section 3.1.1), the presence of calcium spikes does not confer any advantage to the neuron in detecting coincident input shared across the somatic and distal compartments.

The 10/20 setting agrees better with the experimental observations reported in Larkum et al. (1999a), in which a current input at the soma triggers an action potential, which then back propagates to the distal compartment, and in combination with current input at the distal compartment triggers a calcium spike, resulting in a burst of action potentials. Also, bursts of size ≥4 are triggered from correlation values of around 0.3 onwards, demonstrating a more effective cooperativity across compartments than when shared inputs are distributed equally across the compartments. For the rest of the manuscript, we therefore focus on the case that the coincident inputs arrive predominantly at the distal compartment.

#### 3.1.3. Critical frequency in stationary noise

It has previously been demonstrated *in-vitro* that when several step current stimuli, each sufficient to trigger an action potential on its own, are applied to the soma of the layer 5 pyramidal neuron at a sufficiently high frequency (usually between 70 and 100 Hz), a calcium spike is triggered at the distal compartment. This leads to further somatic depolarization and typically further action potentials (Larkum et al., 1999b; Shai et al., 2015). The lowest frequency for which this phenomenon occurs is known as the critical frequency. The back-propagating action potentials sum up supralinearly at the distal compartment when they are evoked at or above the critical frequency due to the active recruitment of voltage gated calcium channels at the distal apical dendrites. Below the critical frequency, the currents at the distal compartment due to the action potentials sum up linearly. As a result, calcium spikes can be triggered without distal inputs. The critical frequency phenomenon is commonly cited as the basis for which synaptic inputs at other parts of the neuron (e.g., basal dendrites) could lower the amount of distal synaptic inputs required to trigger a calcium spike, thus achieving coincidence detection of synaptic inputs across different parts of the neuron (Larkum et al., 1999a,b; Williams and Stuart, 2002).

Our default parametrization of the three compartment neuron model as described in Chua et al. (2015) and Section 2.1 is fitted to reproduce the experimental results presented in Figures 1C–E of Larkum et al. (1999a) and Figures 5C2, 6D of Larkum et al. (2001), and does not exhibit the critical frequency phenomenon. Nonetheless, as shown in Section 3.1.2, coincidence detection across the somatic and distal compartments (shared input) is demonstrated despite the parametrization of the neuron model not reproducing the critical frequency phenomenon. Hence, in contradiction of the claims in Spruston (2008), we conclude that the phenomenon characterized by the critical frequency is sufficient but not necessary for coincidence detection.

**Figure 8:**
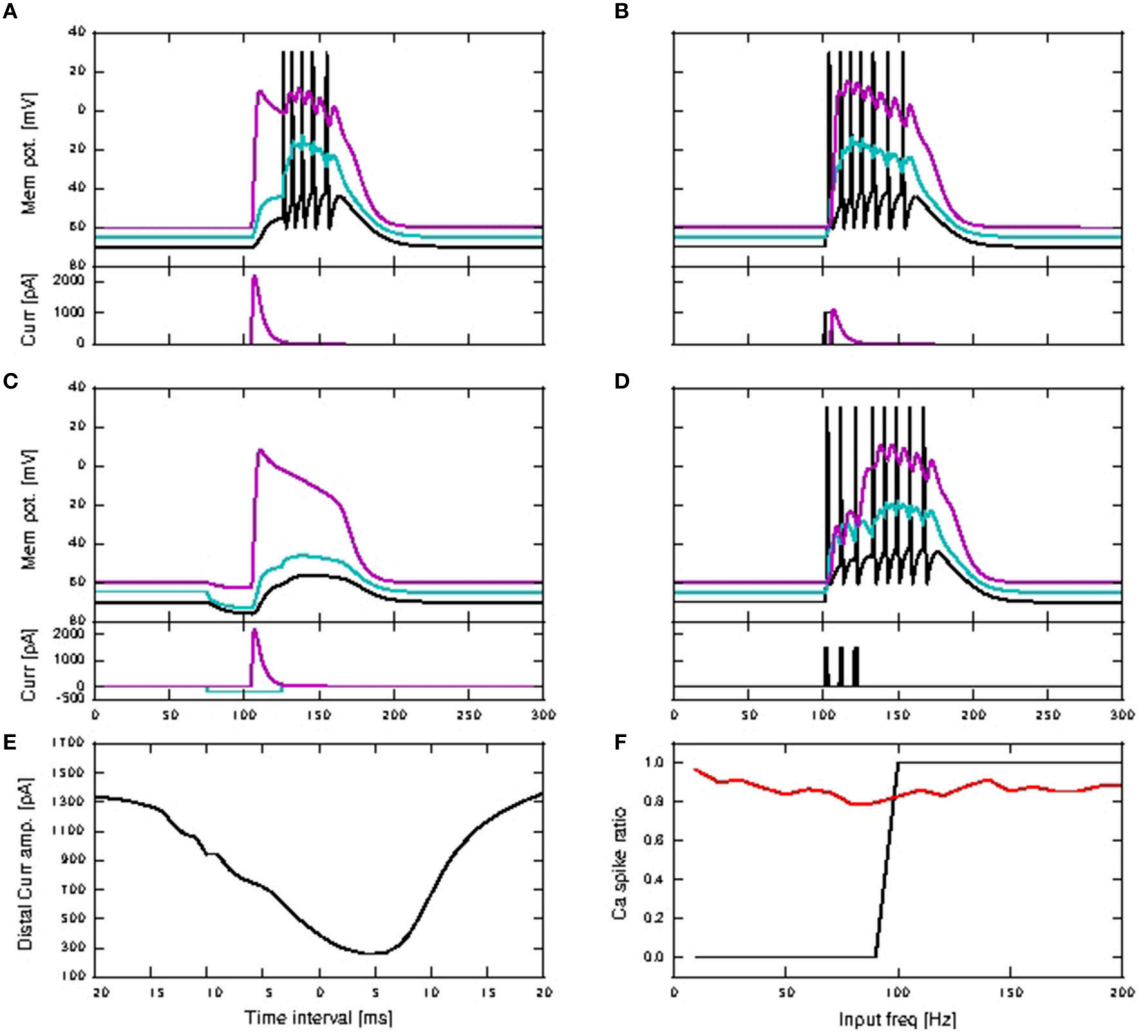
**Simulation results for modified three-compartment model which reproduces the critical frequency property**. **(A–D)** Top panels show the membrane potentials for each compartment against time inms (black: soma, cyan: proximal, magenta: distal), while the lower panels show the corresponding DC stimulation injected at each compartment against time inms. See main text for the stimulation details. **(E)** Minimum amplitude of the beta current at the distal compartment required to trigger a calcium spike when applied together with the somatic step current as in **(B)**. **(F)** Calcium spike ratio (proportion of step current stimuli that trigger a calcium spike) as a function of input frequency for the neuron with modified parameters in the case of no background noise (black) and with background noise (red).

By changing a minimal set of parameters, listed in the final section of Table 1 in the Appendix (Supplementary Material), we are able to reproduce the critical frequency property in our model, with a critical frequency at 100 Hz of stimuli input rate. The response of the neuron model with modified parameters to a variety of input stimuli is demonstrated in Figure 8. In Figure 8A (corresponding to Figure 1E of Larkum et al., 1999a), a beta current, with amplitude 2200 pA and time constants 5.0 and 1.0ms, is applied at the distal compartment, triggering a calcium spike which then propagates to the soma and triggers five action potentials, three more than for the original parametrization. If the same step current is applied again, followed 4ms later by a beta current of half the amplitude, i.e., 1100 pA at the distal compartment (see Figure 1D of Larkum et al., 1999a), this triggers a calcium spike at the distal compartment which then causes six additional action potentials (four more than the original parametrization), as shown in Figure 8B. If, however, 30ms before applying the beta current, a hyper-polarizing step current of −200 pA is applied for 50 ms at the proximal compartment (Figure 5C2 of Larkum et al., 2001), the calcium spike is still triggered but is not able to trigger action potentials at the soma, as shown in Figure 8C. A hyper-polarizing current of −200 pA as used in Figure 6D of Larkum et al. (2001) is enough to prevent triggering of action potentials by the calcium spike, as for the original parametrization. These results demonstrate that the modified parametrization still reproduces the key experimental results reasonably well, albeit not as accurately as our default parametrization.

Figure 8D illustrates the behavior expected of a neuron subject to stimulation above the critical frequency: three step currents, each of amplitude 1500 pA and duration 2 ms, are applied at the somatic compartment with 100 Hz frequency, triggering a calcium spike which triggers another five action potentials. Figure 8E shows the amplitude of the distal current required to trigger a calcium spike when applied in conjunction with a somatic step current. The time interval refers to time of onset of the distal current relative to the somatic current. This agrees qualitatively with Figure 2D of Larkum et al. (1999a). The minimal amplitude of the beta current required to trigger a calcium spike is less than 300 pA, compared with around 1000 pA for the original parametrization. Hence the calcium spike can now be triggered with even fewer distal synaptic inputs when coincident with an action potential, when compared to the original parameters. The critical frequency of the modified neuron model is 100 Hz, as shown in Figure 8F, and so is in agreement with the findings presented in Larkum et al. (1999b) and Shai et al. (2015).

However, repeating the experiment in the presence of background noise (without coincident synaptic inputs; see Section 2.2.2), reveals a very different picture. In this case, calcium spikes are triggered for the whole frequency range, with average of 87%, and minimum of 79% of total stimuli (Figure 8F). The original parametrization results in no calcium spikes across the whole tested frequency range of 10–200 Hz (data not shown). This suggests that the experimentally observed critical frequency property is an artifact of the experimental conditions, in which synaptic noise arrives at only a small fraction of the number of synapses the layer 5 pyramidal neuron typically has (Larkum et al., 1999b; Shai et al., 2015), and so has little relevance for neural information processing.

In the presence of background noise, calcium spikes are triggered by fewer coincident inputs for the modified parametrization than the original, which then results in burst of more action potentials as well. The simulation results in subsequent sections are hence qualitatively similar for both parametrization. As the critical frequency property does not hold in the fluctuation driven regime, we return to the original parametrization for the network investigations in the following section.

### 3.2. Network simulation

The results of the previous section demonstrate in a single neuron setting that coincident inputs are able to trigger calcium spikes and in turn bursts of action potentials: bursts beget bursts. Here, we investigate a possible functional role of this dynamical feature, namely intensifying and stabilizing the propagation of synchronous activity in a FFN consisting of a series of pools of neurons with strong connections from each pool to the next. We examine the behavior of an FFN embedded in a topological recurrent network with a Gaussian connectivity profile, as described in Section 2.2.3 [parameters detailed in Table 4 in the Appendix (Supplementary Material)].

The FFN is constructed from existing neurons and synaptic connections. The neurons in each pool of the FFN are topologically close to each other, and a detailed balance (Vogels and Abbott, 2009) to increase network stability is achieved through scaling of all inter-pool synapses (exc-exc, exc-inh, inh-exc, and inh-inh). Strong stimuli are introduced to the excitatory neurons in the first pool, and we investigate the reliability of activity propagation along the network in dependence on the pool size and the relative strengths of the synaptic connections from one pool to the next compared to a non-FFN exc-exc connection. In the following, the relative strength of the FFN connections is termed the scaling factor. In the shared connectivity setting, pre-synaptic excitatory neurons are connected to post-synaptic excitatory neurons at both the somatic and distal compartments while in the distal connectivity setting, they are connected at only the distal compartments.

#### 3.2.1. Shared connectivity setting

In the shared connectivity setting, the initial stimuli trigger one to two action potentials in each neuron of the first pool of the FFN in the passive case [somatic synaptic inputs, refer to Table 4 in the Appendix (Supplementary Material)], whereas calcium spikes and a burst of action potentials are triggered in the active case (initial stimuli are synaptic inputs shared across the somatic and distal compartment). We first present results for the neuron model calcium dynamics representing the calcium dynamics with first order kinetics, while comparison with the fixed waveform calcium dynamics is presented in Section 3.2.3.

**Figure 9:**
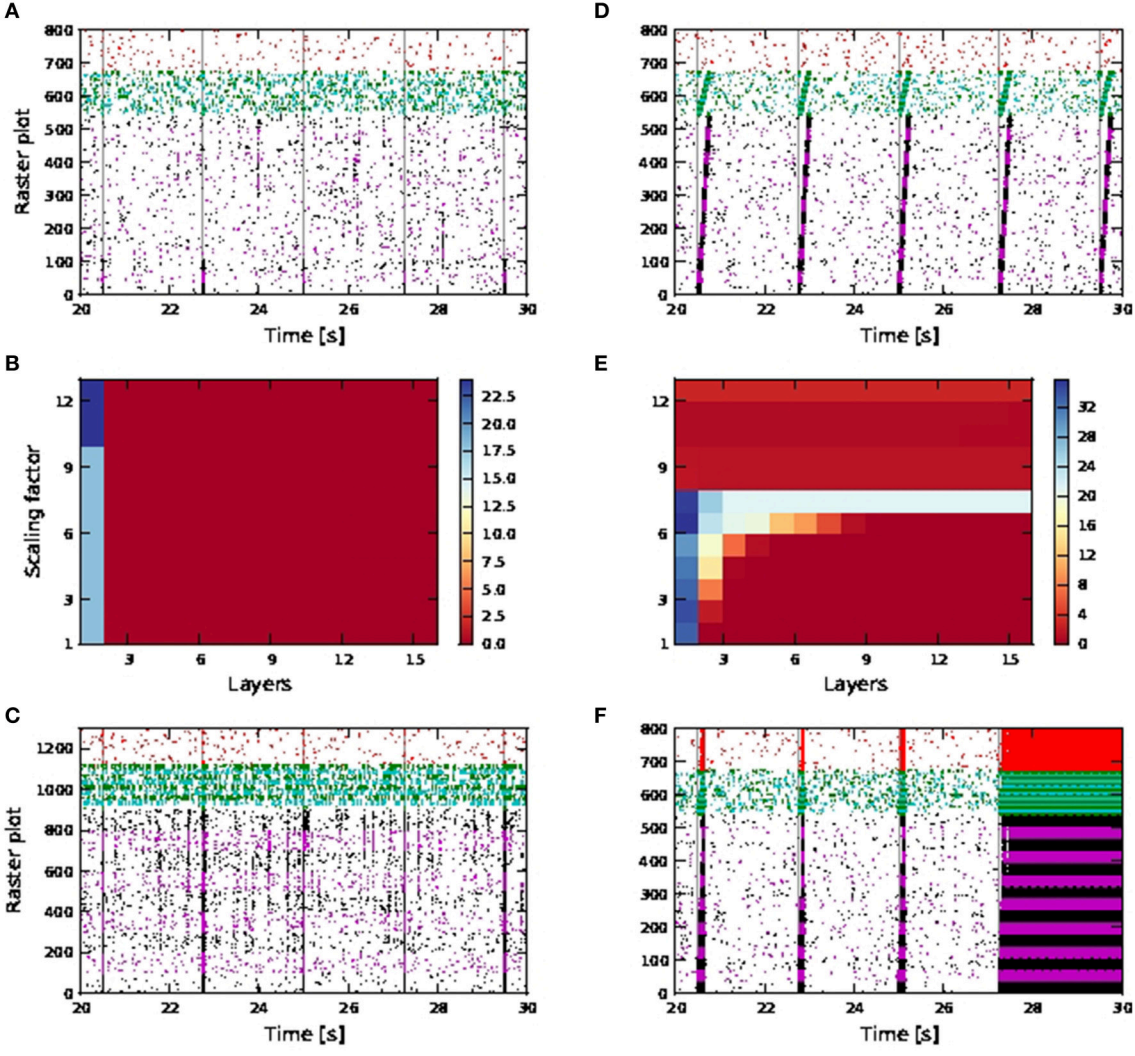
**Activity propagation along a feedforward network for the shared inputs case**. In the raster plots, spikes of neurons in the excitatory FFN are represented by alternate colors of black and magenta, while those of neurons in inhibitory FFN are represented by alternate colors of green and cyan, with 36 and 9 neurons per excitatory and inhibitory pool, unless otherwise stated. Spikes of other randomly selected excitatory neurons are in red. Times of stimuli to the first pool are represented by gray dashed lines. **(A)** Raster plot for the network of passive neurons, with scaling factor of 12. **(B)** Signal-to-noise ratio as a function of the layer of the FFN and the scaling factor for the network of passive neurons. **(C)** Raster plot for passive neurons (with 100 excitatory neurons and 25 inhibitory neurons per pool and scaling factor of 13). **(D)** Raster plot for the network of active neurons, with scaling factor of 7. **(E)** Signal-to-noise ratio as in **(B)** for the network of active neurons. Note different scaling of **(B,E)**. **(F)** Raster plot as in **(D)**, but with scaling factor of 11.

No activity propagation is observed for the passive case for all scaling factors (1−12) as demonstrated in Figures 9A,B. In order to achieve activity propagation, the number of excitatory neurons per pool must be increased from 36 to 100 (Figure 9C). Note that “stripy” spontaneous activities take place even in network of passive neurons. In contrast, a network of active neurons exhibits reliable activity propagation from scaling factor of seven onwards, although the SNR does drop along the length of the network, as shown in Figures 9D,E. However, at high scaling factors, input stimuli can trigger a pathological state whereby all neurons fire continuously and can last for seconds (Figure 9F). Hence activity propagation in active neurons is sensitive to the scaling factor.

**Figure 10:**
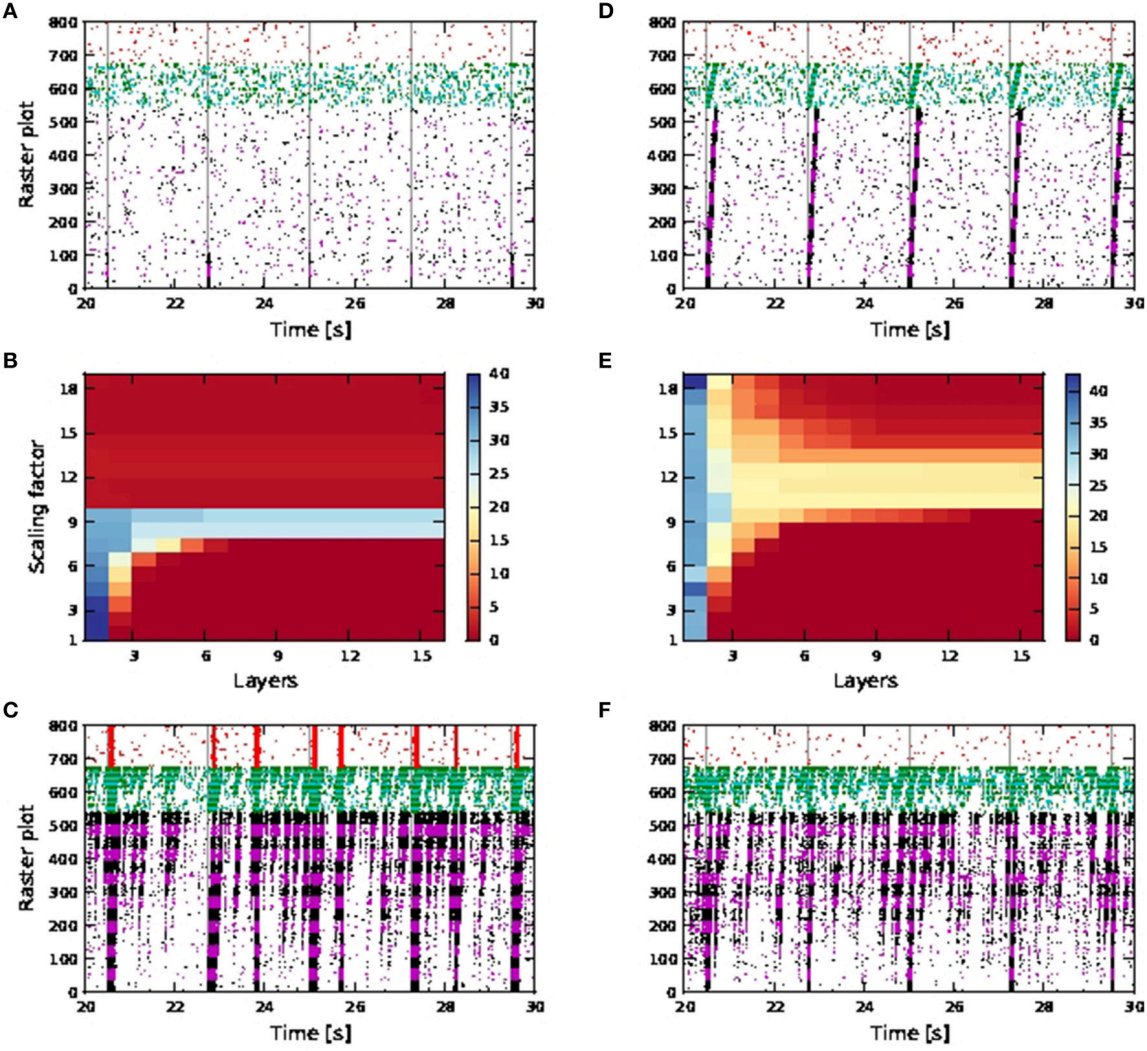
**Activity propagation along a feedforward network for the shared inputs case and increased inhibition (A–C), short term plasticity (depressing) for all exc-exc synapses (D–F) and simultaneous stimuli on first pools of excitatory and inhibitory FFNs**. In raster plot, the spiking activities of neurons in the excitatory FFN are represented by alternate colors of black and magenta, while those of neurons in inhibitory FFN are represented by alternate colors of green and cyan. Spiking activities of other randomly selected excitatory neurons are in red. Time of stimuli are represented by gray dashed lines. **(A)** Raster plot for the network of active neurons, with scaling factor of 8. **(B)** Color-map of SNR for the active case. **(C)** Raster plot for the network of active neurons, with scaling factor of 18. **(D)** Raster plot for the network of active neurons, with scaling factor of 10. **(E)** Color-map of SNR for the active case. **(F)** Raster plot for the network of active neurons, with scaling factor of 18.

In a more inhibitory network, in which the afferent synaptic weights of the inhibitory neurons are increased to 1.7 (excitatory) and −5.2 nS (inhibitory) and synaptic inputs at 90 times the original synaptic weight are introduced at the neurons in the first pool of the inhibitory FFN, simultaneously with the stimuli of the excitatory FFN, network-wide spontaneous activities lasting for seconds are eliminated. Activity propagation is achieved for a bigger range of synaptic scaling factors with better SNR maintained along the length of the network (Figures 10A,B). For larger scaling factors, the entire network is still recruited, albeit for shorter duration: in Figure 10C, strong synchronous firing can be observed in excitatory neurons that are not part of the FFN (marked in red) for periods of tens of milliseconds after each activity propagation event in the FFN. This is in contrast to the lower inhibition case shown in Figure 9F, in which network-wide spontaneous activities can last for seconds. Moreover, stripy spontaneous activities result in a low SNR and make the propagated activities hard to distinguish.

When the synaptic connections between excitatory neurons incorporate depressing short term plasticity [parameters given in Table 4 in the Appendix (Supplementary Material)] without increasing inhibition in the network, spontaneous activities for seconds are also eliminated with high SNR activity propagation achieved for a larger range of synaptic scaling factors (Figures 10D,E). At large scaling factors, stripy spontaneous activities still occur causing SNR of propagated activities to suffer, but spiking activities recruiting the whole network such as those shown in Figure 10C, no longer occur, as shown by the randomly selected excitatory neurons (in red) not firing in synchrony (Figure 10F).

Note that when calcium spikes are triggered by the input stimuli, the stimuli triggered bursts of action potentials in the FFN combine with the spontaneous activities to trigger network-wide activities lasting for seconds. Therefore, increased inhibition or synapses with depressing short term plasticity is needed to keep the network healthy, as demonstrated in Figures 10C,F when such long lasting network activities are not observed even for scaling factor of 18. The network is much more prone to network-wide sustained activities, even at low scaling factors, when afferent synaptic weights of the inhibitory neurons are reduced to 1.2 (excitatory) and −2.8 nS (inhibitory).

Activity propagation is achieved for all scaling factors from 7 onwards Figure 9D, but is highly unstable. At high scaling factors, the propagated activities either end up either recruiting the whole network (for different durations depending on the level of inhibition in the network), or there is too much spontaneous background activities which adversely affect the SNR of the propagated activities (which is the case for increased inhibition and depressing synapses).

The occurrence of spontaneous activities at high scaling factors (such as that shown in Figure 10C), resulting in a drop in SNR is in accordance with the observations of activity propagation by Jahnke et al. (2012). We further observed that with calcium spikes (in contrast to Jahnke et al., 2012), these spontaneous activities can degenerate at higher scaling factors to pathological firing lasting for seconds, as shown in Figure 9F. Such a pathological state can be prevented to a certain extent by introducing depressing short term plasticity between excitatory neurons or increased inhibition in the network (Figure 10).

We further perform single neuron simulations that emulate an excitatory neuron in a FFN, so as to better understand dynamics of activity propagation of an embedded FFN with shared connectivity (data not shown). The network we emulate is the one shown in Figures 10D–F (with depressing synapses). In the shared case, for the active neuron, bursts of size ≥4 are a good coincidence detector for scaling factor from six onwards, with both reliability and informativity at values close to one. For the passive neuron, a single action potential is a good coincidence detector for scaling factor from five onwards, with both reliability and informativity at values close to one. Hence, even without calcium spikes, single action potentials are reliably triggered by coincident inputs. This explains why random fluctuations in the feedforward network that are too weak to trigger a calcium spike may nevertheless lead to spontaneous activities, leading to an increase in background noise and affecting SNR for propagated activities. It is also plausible that the pathological state of firing may be due to increased spontaneous activities during calcium spike mediated activity propagation, which then spreads to the whole network for a sustained period, without coping mechanisms such as short term plasticity and increased network inhibition.

#### 3.2.2. Distal connectivity setting

For distal connectivity, activity propagation is only achieved in the case that calcium spikes are active. As the purely distal stimulation has a weaker effect than the shared somatic and distal stimulation, no activity propagation is observed for either the passive or the active neuron model in an FFN consisting of 15 pools, each containing 36 excitatory neurons with first order kinetics calcium spike and 9 inhibitory neurons, as used above. We therefore increase the number of excitatory and inhibitory neurons per pool to 100 and 25 respectively. As the network size remains unchanged, the number of pools in the FFN is reduced to nine.

**Figure 11:**
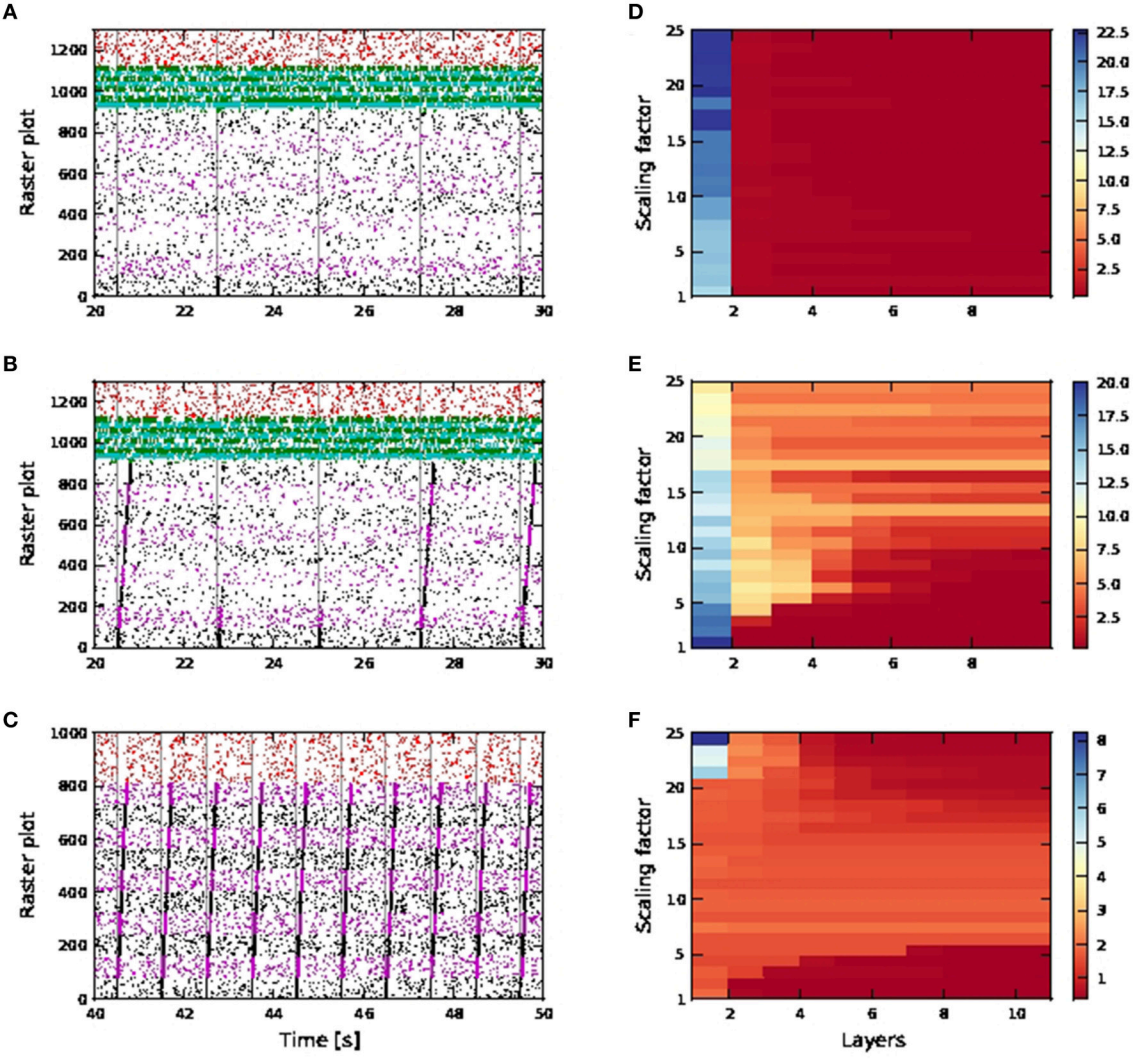
**Activity propagation along a feedforward network for the distal inputs case**. In the raster plots, spiking activities of neurons in the excitatory FFN are represented by alternate colors of black and magenta, while those of neurons in inhibitory FFN are represented by alternate colors of green and cyan. Spiking activities of other randomly selected excitatory neurons are in red. Times of stimuli to the first pool are represented by gray dashed lines. **(A)** Raster plot for the network of passive neurons, with scaling factor of 24. The FFN consists of nine pools, each containing 100 excitatory neurons and 25 inhibitory neurons. Inhibition level as per shared case (Figure 10), with detailed balanced input. **(B)** Raster plot for the network of active neurons, all other parameters as in **(A)**. **(C)** Raster plot for the network of active neurons, with scaling factor of 8. The FFN consists of ten pools, each containing 81 excitatory neurons and around 20 inhibitory neurons. Inhibition level reduced compared to shared case (Figure 10), with only exc-exc inter-pool synapses scaled. **(D–F)** Signal-to-noise ratio corresponding to scenarios **(A–C)** as functions of the layer of the FFN and the scaling factor. Note the differing scales.

Without calcium spikes, activity propagation does not take place even for the broader FFN (Figures 11A,D). This is because distal coincident inputs on their own have little impact at the soma and hardly trigger any action potentials. The high SNR in the first pool is due to the input stimuli being applied to somatic, rather than distal synapses, and so has a much stronger effect on the neuron.

For the active neuron model, activity propagation is achieved from around scaling factor 15 onwards, however not all stimuli to the first pool result in full activity propagation, even for scaling factor 24. (Figures 11B,E). Note that for the active neuron networks, initial stimuli are purely dendritic.

As the network is configured to be in an inhibition dominated regime, we also investigated reducing inhibition in the network to check if activity propagation is established: the afferent synaptic weights of the inhibitory neurons are reduced to 1.2 (excitatory) and −2.8 nS (inhibitory). In addition, only the connections between excitatory neurons in the FFN are subject to the scaling factor. In Figures 11C,F, activity propagation is achieved in the network of active neurons for scaling factor 8 onwards, with a high SNR maintained along the length of the network. The first sign of stripy spontaneous activities occurs at scaling factor 17 and deteriorates to that observed in Figure 10F at scaling factor of 24 (data not shown). Hence, with reduced inhibition, activity propagation is achieved in the network of active neurons which remains robust for a much bigger range of scaling factors (8−16). This is in contrast to the shared input case, which is characterized by strong synchronous activity at much lower scaling factors and activity propagation with good SNR in only a narrow band of scaling factors (Figures 9, 10).

This difference is due to the fact that in the distal network, exc-exc connections are located at the distal compartments. Hence in the excitatory FFN, scaled up distal excitatory inputs on their own (if they do not trigger calcium spikes) are severely attenuated at the soma and hardly trigger any action potentials. Hence, random fluctuation of excitatory inputs in the FFN will result in the stripy spontaneous activities at much higher scaling factors, but will do so at relatively lower scaling factors in the shared case. In addition, during the time course of a calcium spike, excitatory inputs to the soma can result in an action potential, whereas excitatory inputs to the distal have conductances of very low amplitude, given that the compartment membrane potential is close to reversal potential for excitatory synapses, and hence have even less effect on the soma. Hence, while the network with distal connectivity requires more neurons in each pool for activity propagation than the network with shared distal and somatic connectivity, activity propagation is very stable and maintains good SNR even with increasing scaling factors (stripy spontaneous activities do not occur).

The distal connectivity results differ from the shared connectivity results and also from the behavior reported by Jahnke et al. (2012) for two reasons. Firstly, as coincident inputs only arrive at the distal compartment, they have to trigger a calcium spike to elicit any action potentials. However, the threshold for a calcium spike is much higher than that of an action potential, and so no spontaneous synchronous behavior emerges until a high scaling factor of 17. Secondly, as the deactivating function *h* of the calcium dynamics has a fairly long time constant (τ_h_ = 50 ms), increasing stimuli are required to trigger calcium spikes in quick succession with the same amplitude (further discussed in Section 3.2.3). This can be circumvented in the shared case by backpropagating action potentials triggered by inputs on somatic synapses with scaled weights. In the distal case, even with reduced inhibition and scaling factor of 24, spontaneous activities stay within the FFN and do not descend into the pathological state as illustrated in Figure 9F.

Note that when it is the presence of calcium spikes that permits activity propagation (i.e., a network of similarly configured passive neurons exhibits no propagation) then the mechanism is different to the classical mode of propagation in an FFN. As burst firing triggered by calcium spikes can last for tens of milliseconds (50–80ms, as opposed to the passive case, in which spiking activities at each pool last for around 10ms), such a firing mode effectively allows post synaptic neurons in the next pool a bigger integration time window so as to trigger calcium spikes and hence burst firing. As a result, propagation is also slower: compare activity propagation for passive and active FFNs in Figures 9C,D, whereby the last pool of neurons (pool number nine) in the passive case spikes 15ms later after stimulus onset at first pool (100 neurons per pool, scaling factor 13) while the last pool in the active case spikes 200–220ms later after stimulus onset (36 neurons per pool, scaling factor 7).

In the single neuron simulation, we emulate an excitatory neuron in a FFN with distal connectivity as per Figures 11C,F (data not shown). For the active neuron, bursts of size ≥4 are a good coincidence detector for scaling factor from two onwards, with both reliability and informativity at values close to one. For the passive neuron, both reliability and informativity of a single action potential rises gradually with increasing scaling factors, reaching values of approximately 0.4 and 0.6 for reliability and informativity, respectively, at a scaling factor of 25. This is equivalent to all distal excitatory synapses receiving the coincident input, as compared to 15% of distal excitatory synapses (at maximum pairwise correlation value of 0.5) in the original distal single neuron simulation (Figure 4). Hence for the distal case, coincidence detection (and activity propagation) remains highly dependent on calcium spike activation. Without calcium spikes, spiking activities are not reliably triggered, which explains why in the distal case, activity propagation with relatively less noise is achieved for a larger range of weight scales (Figures 11C,F).

#### 3.2.3. Network effect of model calcium dynamics

In this section, we examine the effect of the choice of representation for the calcium dynamics on the behavior of a neuron in an embedded FFN. For the single neuron simulations carried out in Section 3.1 and the network simulations with distal coincident inputs (Section 3.2.2), there is no qualitative difference between the first order kinetics and the fixed waveform approaches.

**Figure 12:**
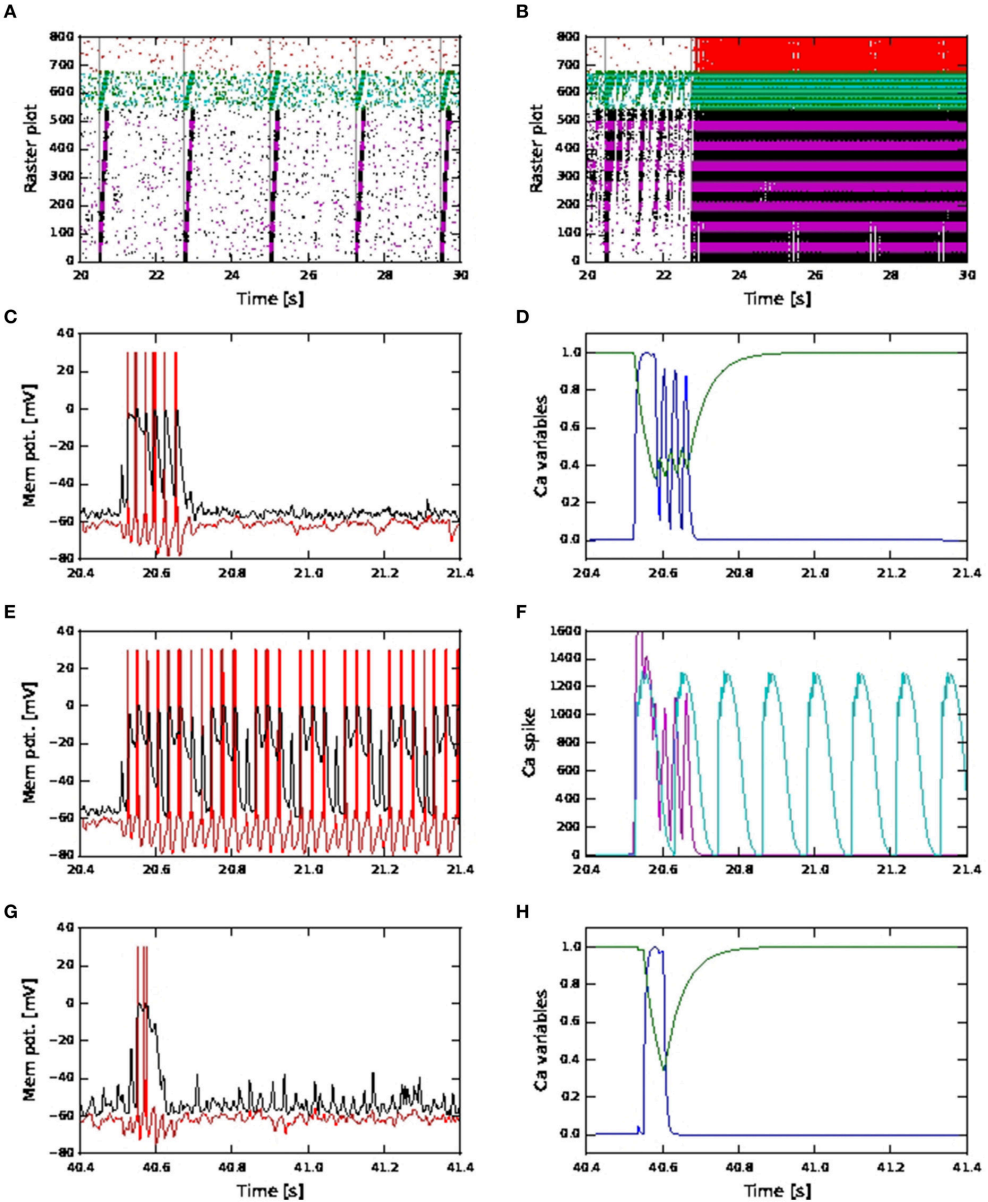
**Network and neuronal responses with calcium dynamics modeled by first order kinetics and fixed waveform**. **(A)** Raster plot for network of active neurons with shared coincident inputs, short term plasticity (depressing) for all exc-exc synapses, scaling factor 10 and calcium spike modeled using fixed waveform. **(B)** As in **(A)**, but with scaling factor 18 and calcium spike modeled using fixed waveform. **(C)** Membrane potential (red: soma, black: distal) for an excitatory neuron in the third pool during activity propagation with shared coincident inputs, increased inhibition, scaling factor 12 and calcium spike modeled using first order kinetics. **(D)** The corresponding plot for the calcium activating (blue) and deactivating (green) functions. **(E)** As in **(C)** but with calcium spike modeled using fixed waveform. **(F)** The corresponding calcium spike (magenta) to the kinetics model **(C)** and calcium spike (cyan) to the fixed waveform model **(E)**. **(G)** As in **(C)** but with distal coincident inputs and decreased inhibition **(H)** The corresponding plot for the calcium activating (blue) and deactivating (green) functions.

However, in the case of shared coincident inputs, this is not so. For a scaling factor of 10, network simulations of shared connectivity using fixed waveform calcium models result in successful activity propagation (Figure 12A, showing results for depressing synapses for Exc-Exc connections). As the scaling factor increases, long continuous firing (as seen in Figure 9F) can be alleviated by increased inhibition or depressing synapses for exc-exc connections (Figures 10C,F) in the kinetics calcium model. However, when the same simulation is repeated with fixed waveform calcium model, the long continuous firing lasting for seconds persists (Figure 12B, showing simulation results for depressing synapses for a scaling factor of 18). The above results for fixed waveform calcium model are the same for increased inhibition (results not shown).

We investigate why this maybe the case by observing the membrane potentials and calcium variables of excitatory neurons in the pools for the above simulations. In Figure 12C, we observe that during activity propagation, one of the excitatory neuron in the third pool first discharge a calcium spike (with three action potentials), followed shortly by another three calcium spikes of smaller amplitudes (with one action potential each). In Figure 12D, the corresponding activating function has one full long peak, followed by three smaller, shorter peaks. Value of the deactivating function goes to around 0.4 during the first calcium spike and remains there during the next three calcium spikes and only fully recovers afterwards. Hence the later three calcium spikes are triggered when the underlying calcium variables have yet to fully recover from the first calcium spike. As such, these later spikes are both smaller in amplitude and shorter in duration. Such calcium spiking activities are observed in other excitatory neurons in different pools during activity propagation. In Figures 12E,F, we observe that in the case of fixed waveform calcium model, calcium spikes, all of the same waveform, are triggered in quick succession, resulting in continuous firing, while the calcium spikes modeled using kinetics lasted for the duration of approximately two calcium spikes (fixed waveform). Hence the long recovery period of the calcium variables in the kinetics model helps to prevent such continuous firing.

In the distal case, as shown in Figures 12G,H, only one calcium spike is triggered during activity propagation, since there is no “stripy” spontaneous activities (present in the shared case) to trigger subsequent calcium spikes. Hence, simulation results from both calcium models in the case distal coincident inputs are qualitatively the same.

The difference in network activities for high scaling factors in the case of shared coincident inputs can therefore be attributed to the different handling of the refractory period. In the kinetics model, the calcium spike does not have a hard refractory period. Subsequent calcium spikes can still be triggered if the membrane potential is high enough, even when the underlying calcium variables have yet to fully recover. These new spikes are smaller while the deactivation variable remains at around the same value. In the fixed waveform model, the calcium spike has a hard refractory period lasting the entire duration of the calcium spike (100 ms), during which no new calcium spike can be triggered. Once the refractory period is over, if the calcium threshold is again reached, a new calcium spike of the same waveform is triggered. Hence, while there can be several more calcium spikes in the same duration emitted by the model using first order kinetics, it is less effective in sustaining continuous firing, due to the relatively longer time constant of the deactivating function (τ_*h*_ = 50 ms).

Hence, from the above observations, the fixed waveform calcium model would give the same results as the kinetics calcium model in simulations in which calcium spikes are not triggered in quick succession, as shown in Figures 12G,H. Given that the fixed waveform model is computationally simpler and thus faster (Chua et al., 2015), we therefore recommend its use in these cases.

## 4. Discussion

In this study, we investigate the effects of calcium spikes on coincidence detection by a single neuron and activity propagation in an embedded feed-forward network, using the three compartment neuron model previously developed in Chua et al. (2015). We show that calcium spikes are necessary for detection of purely distal coincident inputs; without them, no action potentials are triggered. Previous experimental and modeling studies focusing on single neuron dynamics have consistently shown that synaptic inputs at the distal dendrites have to activate the voltage dependent calcium channels in order to propagate to the soma (Larkum et al., 1999b, 2009; Shai et al., 2015). We have extended the above understanding to show that even for large coincident inputs at the distal dendrites, the calcium spike is still required for effective propagation toward the soma and informative and reliable coincidence detection. However, the presence of local NMDA receptors in a biological neuron evoking NMDA spikes, not represented in our model, may reduce the threshold for the amount of synaptic inputs required to activate the calcium channels (Larkum et al., 2009).

Our results also show that calcium spikes trigger large bursts of action potentials for coincident inputs that are shared between the distal and somatic compartments. In the absence of calcium spikes, bursts of size two are triggered instead. Thus, a passive neuron can detect shared coincident inputs, albeit with only a modest change in firing activities. The default parametrization of our neuron model does not reproduce the critical frequency phenomenon, in which current stimuli applied to the soma of the layer V pyramidal neuron at a sufficiently high frequency trigger calcium spikes, which in turn trigger further bursts of action potentials (Larkum et al., 1999b; Shai et al., 2015). However, we demonstrate that a modified parametrization of our neuron model does reproduce the critical property, but that this property is not maintained when embedded in the fluctuation driven regime. Here, somatic input currents trigger bursts of action potentials independent of their frequency. Hence the role of critical frequency *in-vivo* is not clear: whilst it is sufficient for coincidence detection, our results argue against the claim that it is necessary (Spruston, 2008).

To understand the consequences of differing coincidence detection capacities for network activity, we examined the behavior of a feed-forward network embedded in a randomly connected network with a Gaussian connectivity profile. Again we distinguished between a scenario in which the feed-forward connections exist purely at the distal dendrite of the downstream neurons, or at both the distal and the somatic compartments. In the case of exc-exc synapses made solely with the distal dendrite, activity propagation only takes place in the presence of calcium spikes, even when the width of the feed-forward network is increased. In the presence of calcium spikes, activity propagation is stable with respect to increasing the amplitude of the synaptic connections within the network.

If exc-exc synapses are established with both the distal and somatic compartments, the presence of calcium spikes enables activity propagation to take place with much fewer neurons per pool than in the case of passive neurons. However, with both active and passive neurons, as the strength of the recurrent synapses are increased, spontaneous spiking activities spanning several pools of the feed-forward network begin to occur with increasing prevalence, adversely affecting the signal to noise ratio of the activity propagation. In networks of active neurons with strong synapses, the spontaneous activity interacts with the propagated activity to result in network level synchronized spiking, which can last for seconds. Thus, while activity propagation is very robust for active neurons with respect to the strength of synapses, it only results in distinguishable signaling in a much smaller parameter range.

A notable difference between the purely distal and shared connectivity networks is that, with increasing synaptic strength, activity propagation remains stable in the former case (Figure 11), but becomes unstable in the latter (Figure 9). The reason is primarily that synaptic inputs at the distal compartment, even when synapses are strong, do not result in action potentials when there are no large distal coincident inputs to trigger calcium spikes. On the other hand, in the shared case, sufficiently strong random synaptic inputs to the soma result in synchronous spontaneous activities, which combine with calcium spike enabled propagation activities to result in network level sustained activities. The amount of coincident inputs required to trigger a calcium spike is also reduced in the event of an action potential due to its active backpropagation to the distal compartment. Even if we increase the inhibition in the network, synchronous spontaneous activities are still prevalent, although network level sustained activities only last for approximately the duration of the calcium spikes and not for seconds.

This would necessarily imply that if activity propagation is driven by binding (using calcium spikes) of feedforward and feedback inputs as suggested by Larkum (2013), it is a highly unstable process and other neuronal or network mechanism such as short term plasticity (other than increased network inhibition and detailed balance of excitation and inhibition) is needed to keep it in check. It is also in the shared case that the different calcium models lead to different network dynamics at high scaling factors. This is due to the fact that the kinetics model has a much longer recovery period than the duration of a calcium spike before a full strength calcium spike can be triggered again, whereas the refractory period of the fixed waveform model is that of the calcium spike duration. It remains to be seen whether a longer “soft” refractory period, during which lower amplitude calcium spikes can still be triggered, would be able to reproduce the network results of the kinetics model.

Whereas our study makes no claim for the existence of feed-forward networks in the brain, there are nonetheless interesting implications for the large layer 5 pyramidal neurons. The distal tuft dendrites of these neurons receive long range corticocortical and thalamocortical inputs in layer 1 (Kuhn et al., 2008; Petreanu et al., 2009), which in turn then project to superficial layers of other cortical regions (Douglas and Martin, 2004). Our results suggest that these axon-distal connections could be the substrate for reliable propagation of coincident activity.

Activity propagation becoming unstable at higher synaptic scaling factors (> 6.5) was previously observed in Jahnke et al. (2012). We demonstrate that this result is dependent on where coincident input arrives; with purely distal connectivity, activity propagation remains stable for a larger range of scaling factors. To check the validity of our conclusion, we have also run simulations of embedded FFNs using the point neuron model with fast sodium dendritic spikes proposed by Jahnke et al. (2012). We find that with the faster dendritic spike dynamics, we observe somewhat worse activity propagation results as compared to our simulations using shared connectivity settings as shown in Figures 9D,E (see Supplementary Material for details). Our discovery of stable and distinguishable propagation across a range of synaptic strengths that does not result in spontaneous synchronous activity is thus dependent on a dendritic spiking mechanism residing at a compartment electrotonically distant from the soma. This is not achievable with a classical point neuron model, although we do not exclude the possibility of a formulation of a point neuron that generates an effective electrotonic distance. Whereas recent works such as Memmesheimer and Timme (2012) and Wybo et al. (2015) demonstrate that point neuron models can successfully model the somatic effect of non-linear integration of dendritic inputs, our work shows that at the network level, the electrotronic distance in a compartmental model can result in very different network dynamics.

Our three compartment neuron model by no means captures all the properties of calcium spikes, yet it not only allows us to investigate the role of calcium spikes but also potentially how the different operating modes of the pyramidal neuron (integrator vs. coincidence detector) interact with the calcium spike, as one main implication of our findings is that fluctuating synaptic inputs to the somatic compartment are integrated and trigger action potentials (rate coding), whereas synaptic noise at the distal compartment has little effect on the soma, and only large synchronous inputs can trigger calcium spikes to further trigger bursts of action potentials (temporal coding). The concept of different parts of the pyramidal neuron having a different coding scheme is not new, however in Branco and Häusser (2011), the gradient was shown to exist along single dendritic branches, while here the gradient is suggested to be between the soma and distal compartments. However, it would be premature to conclude this on the basis of the current study, as we did not consider other (local) dendritic spikes such as NMDA spikes that could reduce the requirement for large synchronicity in the distal compartment (Larkum et al., 2009). Moreover, bursts of action potential may also be triggered by basal synaptic inputs due to NMDA spikes (Polsky et al., 2004). In such a case, calcium spikes at the distal dendrite are not involved, and basal/somatic coincident inputs and bursts of action potentials as outputs are then mediated by a different dendritic mechanism.

A further restriction of scope in our study is that we did not take into account how calcium spikes may be hijacked by distal or proximal inhibitory inputs so as to fail in triggering bursts of action potentials. Such a mechanism maybe mediated by the Martinotti cells,which upon receipt of bursts of action potentials from a pre-synaptic pyramidal neuron then laterally inhibits neighboring pyramidal neurons (Silberberg and Markram, 2007). This could then implement a winner-takes-all network. During the time course of a calcium spike, the distal membrane potential is near to the excitatory reversal potential. As such excitatory synaptic inputs during this period results in very little conductances and is as good as lost.

Despite this limitation, our results illustrate the critical role calcium spikes play in influencing the spiking activities of individual neurons and networks of neurons. When inputs are asynchronous, a network of neurons with active calcium dynamics behaves like a network of passive neurons. However, in the event that a spatially close sub-population of these neurons receives coincident inputs, the network dynamics can change fundamentally, generating activity propagation or sustained network spiking as shown in Figures 9D,F. We know that biological neurons, especially pyramidal cells, do spike dendritically. While previous experimental recordings lend much support to the notion these biological neural networks behave as in the asynchronous irregular regime (Brunel, 2000), this may change as soon as coincident inputs arrive, for instance due to a sensory stimulus. Such a stimulus could then trigger large feedforward burst of action potentials targeting spatially close neurons in the sensory receptive field, further triggering a cascade of events, made possible by dendritic spikes. Already, there are experimental findings showing dendritic spikes are critical for neuronal functions *in-vivo* (Sivyer and Williams, 2013; Smith et al., 2013; Grienberger et al., 2014; Palmer et al., 2014). Hence, if we are to study and model how cortical networks process stimuli and attentional feedback, we have to take into account how dendritic spikes fundamentally change network behavior in the presence of synchrony.

## Author contributions

The work presented here was carried out in collaboration between all authors. YC and AM designed the research; YC performed the simulation; YC and AM analyzed the results; YC and AM wrote the paper. All authors have read and approved the final published manuscript.

### Conflict of interest statement

The authors declare that the research was conducted in the absence of any commercial or financial relationships that could be construed as a potential conflict of interest.
