# Supplementary material for: Effects of Calcium Spikes in the Layer 5 Pyramidal Neuron on Coincidence Detection and Activity Propagation
Source: Front Comput Neurosci. 2016 Jul 22;10:76. doi: 10.3389/fncom.2016.00076 (PMC4957534; doi:10.3389/fncom.2016.00076)
Supplement: Supplementary file 1 [file DataSheet1.pdf]

Chapter. 3 Supplementary Materials

In order to compare the effect on network behavior of slow calcium spikes to that of fast sodium dendritic spikes, we have implemented the neuron model presented by Jahnke et al. (2012) and created networks with embedded FFNs as detailed in the main manuscript, Sec. 2.2.3. As the connectivity probability used in Jahnke et al. (2012) is 0.03 while we use 0.1, we ran two sets of simulations with slightly different network parameters for comparison and completeness.

In the first setting, with connectivity probability of 0.03, we use the same synaptic weights, input currents and Poisson input firing rates as described by Jahnke et al. (2012). As before, no activity propagation is achieved in the passive case, as shown in Fig. 1a,b. Successful activity propagation in the active neuron network for some of the stimuli starts from scaling factor of 8 onwards (Fig. 1c), as do stripy spontaneous activities. Fig. 1d shows that successful activity propagation is achieved for all five stimuli from scaling factor of 10 onwards. In scaling factors of 10 and above, spontaneous activities occupy the entire network, not just pertaining to the FFN. Some of the propagated activities at scaling factor of 10 onwards also occupy the entire network, which is not desirable. Fig. 1e shows the SNR for the active neurons, with successful activity propagation for scaling factors $7 - 10$. However, this fails to illustrate that for some of the propagated activities, the entire network is recruited to fire synchronously. Fig. 1f shows the raster plot for the network with scaling factor of 12, in which stripy spontaneous activities are frequent and recruit the entire network.

In the second setting, we increase the connectivity probability to 0.1 and make several changes to the network parameters so as to keep the active neuron network in the asynchronous irregular firing regime for a scaling factor of 1. The excitatory-excitatory synaptic weight is reduced to 0.5 nS from 0.6 nS, and the excitatory-inhibitory weight is increased to 0.65 nS. The inhibitory-excitatory and inhibitory-inhibitory synaptic weights are scaled accordingly so as to keep the mean membrane potentials of both excitatory and inhibitory neurons near to the spiking threshold.

As for the first setting, Fig. 2a,b demonstrate that there is no activity propagation for the passive case. Successful activity propagation for some stimuli start from scaling factor of 2 onwards but as demonstrated in Fig. 2c,d (scaling factors of 6 and 8 respectively), some of these propagated activities end up recruiting the whole network. As seen from Fig. 2d, stripy spontaneous activities occupying the whole network occur as early as scaling factor of 8. The SNR measurements displayed in Fig. 2e suggest that successful activity propagation with good SNR occurs for scaling factors $7 - 8$, but the individual raster plots reveal that successful activity propagation for all stimuli starts only from scaling factor of around 10 onwards. However, as shown in Fig. 2f, at scaling factor of 10, stripy spontaneous activities are so frequent that it becomes hard to distinguish propagated activities from noise.

Hence, while activity propagation for some stimuli start from different scaling factors for the different network configurations, successful activity propagation for all stimuli start from scaling factor of 10 for both networks. At this scaling factor, the background noise due to stripy spontaneous activities has become quite frequent. Also, in both networks, propagated activities, if successful, often end up recruiting the whole network.

The simulations carried out with a neuron model incorporating a fast sodium spike show that success of activity propagation with good SNR is only moderate, and successful activity propagation for all stimuli occur at rather high scaling factors when stripy spontaneous activities are frequent. Moreover, propagated activities often recruit the entire network. The above two observations stand in stark contrast to the case of the multi-compartment neuron model with calcium spike. In the shared

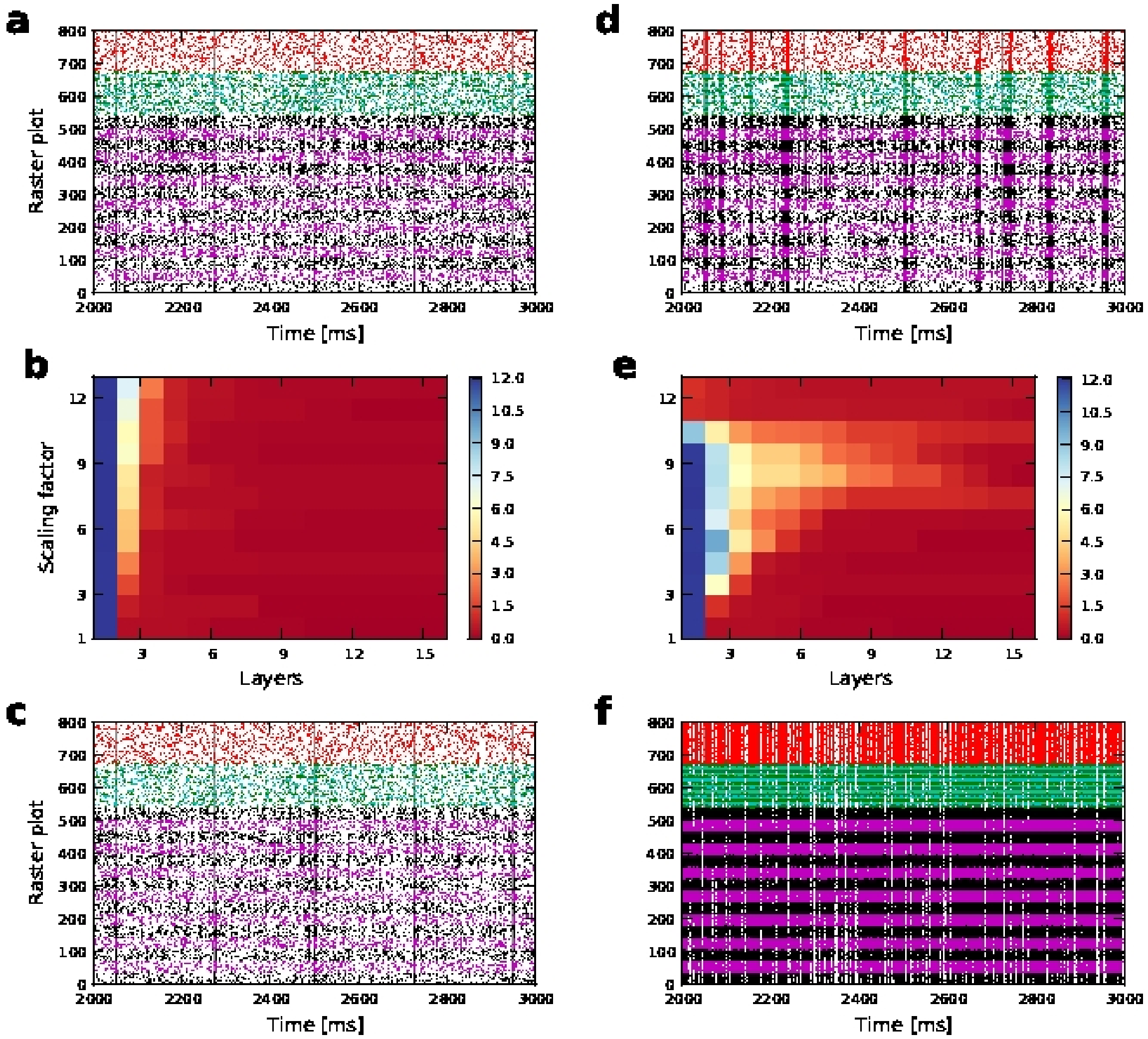

Figure 1: Activity propagation along a feedforward network for neuron models with fast sodium dendritic spikes, and connection probability of 0.03. In the raster plots, spikes of neurons in the excitatory FFN are represented by alternate colors of black and magenta, while those of neurons in inhibitory FFN are represented by alternate colors of green and cyan, with 36 and 9 neurons per excitatory and inhibitory pool. Spikes of other randomly selected excitatory neurons are in red. Times of stimuli to the first pool are represented by gray dashed lines. (a) Raster plot for the network of passive neurons, with scaling factor of 12. (b) Signal-to-noise ratio as a function of the layer of the FFN and the scaling factor for the network of passive neurons. (c) Raster plot for the network of active neurons, with scaling factor of 8. (d) Raster plot as in (c), but with scaling factor of 10. (e) Signal-to-noise ratio as in (b) for the network of active neurons. (f) Raster plot as in (d), but with scaling factor of 12.

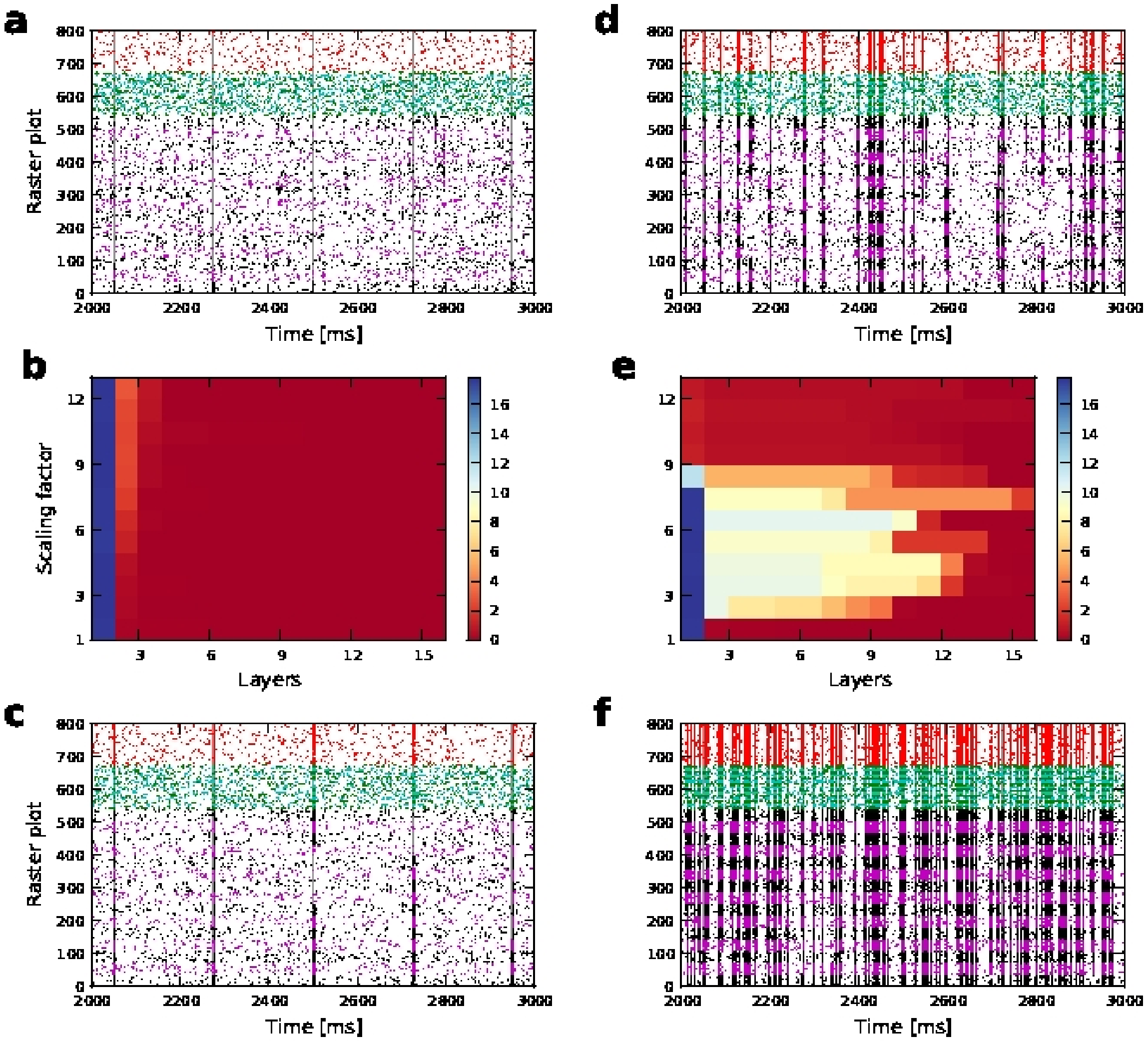

Figure 2: Activity propagation along a feedforward network for neuron models with fast sodium dendritic spikes, and connection probability of 0.1. In the raster plots, spikes of neurons in the excitatory FFN are represented by alternate colors of black and magenta, while those of neurons in inhibitory FFN are represented by alternate colors of green and cyan, with 36 and 9 neurons per excitatory and inhibitory pool. Spikes of other randomly selected excitatory neurons are in red. Times of stimuli to the first pool are represented by gray dashed lines. (a) Raster plot for the network of passive neurons, with scaling factor of 12. (b) Signal-to-noise ratio as a function of the layer of the FFN and the scaling factor for the network of passive neurons. (c) Raster plot for the network of active neurons, with scaling factor of 6. (d) Raster plot as in (c), but with scaling factor of 8. (e) Signal-to-noise ratio as in (b) for the network of active neurons. (f) Raster plot as in (c), but with scaling factor of 10.

case, with comparable amount of network inhibition, successful activity propagation with good SNR is achieved, as shown in Fig. 9d,e. In the distal case, successful activity propagation with good SNR is achieved for an even broader range of scaling factors, despite less network inhibition (Fig. 11).

References

Jahnke, S., Timme, M., & Memmesheimer, R.-M. (2012). Guiding synchrony through random networks. *Physical Review X 2*(4), 041016.
