# Supplementary material for: Effects of Calcium Spikes in the Layer 5 Pyramidal Neuron on Coincidence Detection and Activity Propagation
Source: Front Comput Neurosci. 2016 Jul 22;10:76. doi: 10.3389/fncom.2016.00076 (PMC4957534; doi:10.3389/fncom.2016.00076)
Supplement: Supplementary file 2 [file DataSheet2.pdf]

Appendix

Neuron parameters

Table 1: Parameters of the neuron models. Calcium spike parameters for the first model using first order kinetics are listed. The action potential is modeled by a jump in membrane potential and a large leak during refractory period while the threshold is adaptive. Alpha currents are added at the proximal and distal compartment 1 and 2 ms after spike to emulate a back-propagating action potential. Neuron parameters adapted to reproduce critical frequency property is listed at bottom of table.

$\tau_{\mathrm{i}}^{\mathrm{d}}$	inhibitory synaptic time constant	2.0 ms
Neuron parameters (without critical frequency)		
Θ_{base}	base spike threshold	−55.0 mV
t_{ref}	refractory period	2.0 ms
V_{peak}	somatic membrane potential upon spike	30.0 mV
Θ_{+}	adaptive threshold jump	6.0 mV
τ_{th}	adaptive threshold time constant	7.0 ms
g_{sp}	leak across soma-proximal	30.0 nS
g_{pd}	leak across proximal-distal	10.0 nS
Calcium spike parameters		
U_{ca}	calcium reversal potential	30.0 mV
g_{ca}	calcium conductance	70.0 nS
τ_{m}	activating function time constant	5.0 ms
τ_{h}	deactivating function time constant	50.0 ms
m_{half}	activating function half voltage	−21.0 mV
h_{half}	deactivating function half voltage	−24.0 mV
m_{slope}	slope of activating function	0.5
h_{slope}	slope of deactivating function	−0.5
soma parameters		
$g_{\mathrm{l}}^{\mathrm{s}}$	leak during refractory period	150.0 nS
t_{ref}	duration of refractory period	2.0 ms
$g_{\mathrm{l}}^{\mathrm{s}}$	leak	10.0 nS
C^{s}	capacitance	150.0 pF
U_{l}^{s}	resting potential	−70.0 mV
$U_{\mathrm{e}}^{\mathrm{s}}$	excitatory reversal potential	0.0 mV
$U_{\mathrm{i}}^{\mathrm{s}}$	inhibitory reversal potential	−85.0 mV
$\tau_{\mathrm{e}}^{\mathrm{s}}$	excitatory synaptic time constant	1.0 ms
$\tau_{\mathrm{i}}^{\mathrm{s}}$	inhibitory synaptic time constant	2.0 ms
proximal parameters		
$g_{\mathrm{l}}^{\mathrm{p}}$	leak	10.0 nS

Table 1: Parameters of the neuron models. Calcium spike parameters for the first model using first order kinetics are listed. The action potential is modeled by a jump in membrane potential and a large leak during refractory period while the threshold is adaptive. Alpha currents are added at the proximal and distal compartment 1 and 2 ms after spike to emulate a back-propagating action potential. Neuron parameters adapted to reproduce critical frequency property is listed at bottom of table.

$\tau_{\mathrm{i}}^{\mathrm{d}}$	inhibitory synaptic time constant	2.0 ms
C^{p}	capacitance	80.0 pF
$U_{\mathrm{l}}^{\mathrm{p}}$	resting potential	−65.0 mV
$U_{\mathrm{e}}^{\mathrm{p}}$	excitatory reversal potential	0.0 mV
$U_{\mathrm{i}}^{\mathrm{p}}$	inhibitory reversal potential	−85.0 mV
$\tau_{\mathrm{AP}}^{\mathrm{p}}$	time constant of bpAP alpha current during spike	1.0 ms
$J_{\mathrm{AP}}^{\mathrm{p}}$	amplitude of bpAP alpha current	400.0 pA
$\tau_{\mathrm{e}}^{\mathrm{p}}$	excitatory synaptic time constant	1.0 ms
$\tau_{\mathrm{i}}^{\mathrm{p}}$	inhibitory synaptic time constant	2.0 ms
distal parameters		
$g_{\mathrm{l}}^{\mathrm{d}}$	leak	20.0 nS
C^{d}	capacitance	60.0 pF
$U_{\mathrm{l}}^{\mathrm{d}}$	resting potential	−60.0 mV
$U_{\mathrm{e}}^{\mathrm{d}}$	excitatory reversal potential	0.0 mV
$U_{\mathrm{i}}^{\mathrm{d}}$	inhibitory reversal potential	−85.0 mV
$\tau_{\mathrm{AP}}^{\mathrm{d}}$	time constant of bpAP alpha current during spike	1.0 ms
$J_{\mathrm{AP}}^{\mathrm{d}}$	amplitude of bpAP alpha current	310.0 pA
$\tau_{\mathrm{e}}^{\mathrm{d}}$	excitatory synaptic time constant	1.0 ms
Neuron parameters adapted to reproduce critical frequency		
Θ_{+}	adaptive threshold jump	12.0 mV
τ_{th}	adaptive threshold time constant	13.0 ms
τ_{AP}	time constant of bpAP alpha current during spike	2.0 ms
$J_{\mathrm{AP}}^{\mathrm{p}}$	amplitude of proximal bpAP alpha current	1080.0 pA
$J_{\mathrm{AP}}^{\mathrm{d}}$	amplitude of distal bpAP alpha current	837.0 pA

Stimulation parameters for coincidence detection

Input details	
# excitatory synapses per compartment	2000
# inhibitory synapses per compartment	500
Synaptic delay	1.0 ms
Rate of total inputs per synapse	1.0 spikes/s
Rate of mother process	1.0 spikes/s
Copy probability (pair-wise correlation)	$0.01 - 0.5$
Distal coincident inputs (30% of distal excitatory synapses)	
calcium threshold	-21.4 mV
identical synaptic weights	excitatory: 0.6 nS, inhibitory: 1.0 nS
lognormal synaptic weight distribution	excitatory mean: 0.4 nS, inhibitory mean: 0.4 nS
Soma + distal coincident inputs (10%, 20% of distal, somatic excitatory synapses)	
calcium threshold	-23.6 mV
identical synaptic weights	excitatory: 0.6 nS, inhibitory: 1.0 nS
lognormal synaptic weight distribution	excitatory mean: 0.4 nS, inhibitory mean: 0.4 nS

Table 2: Stimulation parameters for the single neuron in background noise with occasional coincident inputs. Coincident inputs are drawn from a mother process Kuhn et al. (2004) with a specified copy probability and impinge on either 30% of the excitatory synapses in the distal compartment, or 10% of excitatory synapses in the soma and 20% of excitatory synapses in distal compartment, see **??** for details. Simulations are carried out for four different scenarios, in which coincident inputs arrive only at the distal compartment or at both somatic and distal compartments, and synaptic weights are identical or lognormally distributed. In each scenario, the synaptic weights are varied accordingly so as to maintain a firing rate of approximately 1 spikes/s and a mean somatic membrane potential of -60 mV in the case of no coincident inputs.

Input details	
# excitatory synapses per compartment	1000
# inhibitory synapses per compartment	250
Synaptic delay	1.0 ms
Distal coincident inputs	
identical synaptic weights	excitatory: 1.0 nS, inhibitory: 1.2 nS
Poisson excitatory rates	1000 spikes/s, 1000 spikes/s,<br>$190 * 1$ spikes/s + $810 * 0.8$ spikes/s
Poisson inhibitory rates	$202.5 * 1.9$ spikes/s + $47.5 * 1$ spikes/s,<br>250 spikes/s, 250 spikes/s
Coincident inputs (with static synapses)	excitatory input, weight :<br>distal-$1 * 40.5 * scale$ nS
Soma + distal coincident inputs	
identical synaptic weights	excitatory: 1.0 nS, inhibitory: 1.2 nS
Poisson excitatory rates	$724.6 * 1$ spikes/s + $275.4 * 0.3$ spikes/s,<br>1000 spikes/s,<br>$465.4 * 1$ spikes/s + $534.6 * 0.3$ spikes/s
Poisson inhibitory rates	$202.5 * 1.2 + 47.5 * 1$ spikes/s,<br>250 spikes/s, 250 spikes/s
Coincident inputs (with depressing synapses)	excitatory input, weight :<br>soma-$1 * 18 * 0.34 * scale$ nS,<br>distal-$1 * 18 * 0.66 * scale$ nS<br>inhibitory input, weight :<br>soma-$1.2 * 4.5 * scale$ nS

Table 3: Stimulation parameters for the single neuron in background noise with occasional coincident inputs, to emulate neuron in feedforward network. Poisson firing rates are stated for the soma, proximal and distal compartments, in that order. For the distally connected network, average firing rates, empirically obtained, for the excitatory and inhibitory neurons are respectively 0.8 spikes/s and 1.9 spikes/s, while the firing rates for the network with shared connectivity are 0.3 spikes/s and 1.2 spikes/s. It is assumed that on average, each coincident input due to propagated activities involves 40.5 (half of pool size and based on connectivity with Gaussian profile) pre-synaptic neurons from the prior excitatory pool in the distal case. As for the shared case, excitatory coincident input involves 18 neurons from the prior excitatory pool, with synaptic weights split across the somatic and distal compartment with ratio of 0.34 : 0.66. In addition there is also the coincident somatic inhibitory input received from, on average, 4.5 neurons from the prior inhibitory pool (detailed balance). Scale here refers to the scaling factor used in network simulations.

Network simulation parameters

Table 4: Network simulation parameters. The network is a balanced random network with neurons regularly spaced on a 1×1 grid. Synaptic weights are selected such for a self-consistent firing rate of around 1 spikes/s and an average somatic membrane potential of around -60 mV. Parameters for shared connectivity are the same as distal unless otherwise stated.

excitatory neurons	8100 ($90 * 90$) three-compartment LIF neurons with conductance synapses on 1×1 layer
inhibitory neurons	2025 ($45 * 45$) LIF neurons with conductance synapses on 1×1 layer
Synaptic delay	1.0 ms
distal connectivity :	
synaptic weights (exc neurons)	excitatory : 1.0 nS, inhibitory : 1.2 nS
excitatory noise (exc neurons)	Poisson spike trains: 1000 spikes/s, 1000 spikes/s, 190 spikes/s at somatic, proximal, distal compartments
inhibitory noise (exc neurons)	Poisson spike trains at 47 spikes/s, 250 spikes/s, 250 spikes/s at somatic, proximal, distal compartments
synaptic weights (inh neurons)	excitatory : 1.5 nS, inhibitory : -4.3 nS
excitatory noise (inh neurons)	Poisson spike trains: 190 spikes/s
inhibitory noise (inh neurons)	Poisson spike trains: 47 spikes/s
exc-exc connections	distal compartment of 810 random exc neurons, with Gaussian connectivity profile, $\sigma = 0.2$
exc-inh connections	203 random inh neurons, with Gaussian connectivity profile, $\sigma = 0.2$
inh-exc connections	somatic compartment of 810 random exc neurons, with Gaussian connectivity profile, $\sigma = 0.2$
inh-inh connections	203 random inh neurons, with Gaussian connectivity profile, $\sigma = 0.2$
stimulus of first pool of FFN (passive)	synaptic input to each exc neuron: 90×1.0 nS at somatic compartment
stimulus of first pool of FFN (active)	synaptic input to each exc neuron: 90×1.0 nS at distal compartment

shared connectivity :	
excitatory noise (exc neurons)	Poisson spike trains at 724.6 spikes/s, 1000 spikes/s, 465.4 spikes/s at somatic, proximal, distal compartments
exc-exc synaptic weights	distal compartment: 0.66 nS, somatic compartment: 0.34 nS
stimulus of first pool of FFN (passive)	synaptic input to each exc neuron: 90×0.34 nS at somatic compartment
stimulus of first pool of FFN (active)	synaptic input to each exc neuron: 30×0.34 nS at somatic, and 60×0.66 nS at distal compartment
synapses with short term plasticity:	unit increment : 1.0, probability of release : 1.0, $\tau_{\mathrm{rec}} = 10\,\mathrm{ms}$, $\tau_{\mathrm{fac}} = 0\,\mathrm{ms}$

References

Kuhn, A., Aertsen, A., & Rotter, S. (2004). Neuronal integration of synaptic input in the fluctuation-driven regime. *J. Neurosci. 24*(10), 2345–2356.
